# The novel antitumor compound clinopodiside A induces cytotoxicity *via* autophagy mediated by the signaling of BLK and RasGRP2 in T24 bladder cancer cells

**DOI:** 10.3389/fphar.2022.982860

**Published:** 2022-09-19

**Authors:** Rong Sheng Zhou, Ji Zhong Zhao, Li Ming Guo, Jia Li Guo, Aida El Makawy, Zong Yun Li, Shao Chin Lee

**Affiliations:** ^1^ Institute of Biomedical Sciences and School of Life Sciences, Jiangsu Normal University, Xuzhou, Jiangsu, China; ^2^ Department of Urology, Xuzhou Central Hospital, Xuzhou, Jiangsu, China; ^3^ Tianjin Saier Biotech, Tianjin, China; ^4^ Cell Biology Department, Biotechnology Research Institute, National Research Centre, Dokki, Egypt

**Keywords:** clinopodiside A, autophagy, apoptosis, BLK, RasGRP2, combinational effect

## Abstract

In the study, we investigated the anti-cancer effect of clinopodiside A and the underlying mechanisms using T24 bladder cancer cells as an experimental model. We found that the compound inhibited the growth of the bladder cancer cells *in vitro* and *in vivo* in a in a concentration- and dose-dependent manner, respectively, which showed a combinational effect when used together with cisplatin. In the bladder cancer cells, clinopodiside A caused autophagy, which was mediated by the signaling of BLK and RasGRP2, independently. Inhibition of the autophagy by chemical inhibitor 3-methyladenine or by the inhibition of the signaling molecules attenuated the cytotoxicity of clinopodiside A. Further analyses showed that clinopodiside A acted in synergism with cisplatin which itself could trigger both autophagy and apoptosis, which occurred with concomitant enhancements in autophagy and the cisplatin-evoked apoptosis. In conclusion, our results suggest that clinopodiside A inhibits the growth of the bladder cancer cells *via* BLK- and RasGRP2-mediated autophagy. The synergistic effect between clinopodiside A and cisplatin is attributed to the increases in autophagy and autophagy-promoted apoptosis. Clinopodiside A is a promising investigational drug for the treatment of cancer, at least blabber, which can be used alone or in combination with clinical drug(s).

## 1 Introduction

Cancer is a common disease and is the second leading cause of death globally. According to the International Agency for Research on Cancer, in 2020, there were 19.3 million cancer patients diagnosed and 10 million cancer deaths worldwide. It is estimated that the number cancer patients might reach 28.4 million in 2040, with a 47% increase from 2020 (Sung et al., 2021). This imposes a significant burden on the global healthcare system and the economy. In United States alone, the cost of cancer treatment has increased by 26% from 124 billion dollars in 2010 to 157 billion dollars in 2020, and the cost is expected to continue to grow by 5.6 percent each year for the following decade (Mariotto et al., 2011). More specifically, bladder cancer is the most common malignancy of the urinary tract and one of the most prevalent cancers worldwide (Dobruch and Oszczudłowski, 2021). In 2020, the number of bladder cancer cases and fatalities were 573,278 and 212,536, respectively (Sung et al., 2021). The costs of bladder cancer treatment are high, with economic models estimating costs of more than $6 billion by 2020 (Leow et al., 2018).

Despite that surgery, chemotherapy and radiotherapy are common intervention strategies for cancer therapy (Tohme et al., 2017), the treatments have limitations. Surgery can only be used for solid tumor. Moreover, there is window time when is best or suitable for surgery, which patients often miss out (Wyld et al., 2015), as they are diagnosed at an advanced stage (Lee et al., 2018). Even worse is that surgery might promote cancer migration and thus progression (Murthy et al., 1989). Radiotherapy can cause damages to normal tissues, which can even result in functional loss of organs, and is not suitable for advanced cancers (Baskar et al., 2012). Chemotherapy primarily works by interfering with DNA synthesis and mitosis, resulting in the death of rapidly growing and dividing cancer cells. The agents are nonselective and can cause severe unintended and undesirable side effects by damaging healthy tissues. Indeed, chemotherapeutic drugs’ severe side effects on healthy tissues and organs are a major contributor to cancer patients’ high mortality rates (Senapati et al., 2018).

In light of this, more selective active compounds with fewer side effects, lower costs, more medicinal properties, and a minimum level of disease resistance have been enlisted as a major significant attribute necessary for cancer treatment, particularly from biological and natural sources (Newman and Cragg, 2020). Nature products provide a huge resource for drug development. From 1981 to 2019, 60% of the drugs approved by FDA are developed from nature products or their derivatives. Specifically, before 2016, 77% of cancer chemotherapeutic agents are from or derived from natural compounds (Newman and Cragg, 2020). Although the development of targeted therapy has made significant achievements, which attract great attention at around 1990’s when the development of cancer chemotherapeutic drugs from natural products was basically suspended, the striking drug resistance that commonly occur within short time of targeted therapy largely limits the use of targeted therapy. Therefore, the natural products have returned to the center stage of the anti-cancer drug development from 2000 (Blagosklonny, 2005). Natural products have a high level of biological tolerance, far fewer toxic side effects on the body than clinical chemotherapeutic drugs, as well as a lower rate of drug resistance and are capable of reversing drug resistance (Wang et al., 2015; Yuan et al., 2017).

Clinopodium L. belongs to the Labiatae family and includes 20 species, eleven species of them are in China. For thousands of years, plants in this genus have been widely used in traditional Chinese medicine. *C. chinense* is a widely distributed species in southwest China that has traditionally been used in ethnic medicine to treat colds, hepatitis, enteritis, dysentery, mumps, mastitis, allergic dermatitis, and conjunctivitis (Minru and Yi, 2016). Zhu et al. (2018) reported that the aerial parts of *Clinopodium chinense* (Benth.) O. Kuntze yielded four new ursane-type triterpenoid saponins, clinopoursaponins A–D, six new oleanane-type triterpenoid saponins, clinopodiside VII–XII, and eight known triterpene analogues. Compounds were tested for their ability to protect H9c2 cells from anoxia/reoxygenation-induced apoptosis as well as their cytotoxicity against the 4T1 murine mammary carcinoma cell line. Clinopodiside was found to be protective, while clinopoursaponins A was found to be cytotoxic, with an IC50 value of 7.4 m compared to 7.6 m for the positive control 10-hydroxycamptothecin.

In our latest pilot screening, we obtained preliminary evidence for an inhibitory effect of clinopodiside A (C_48_H_78_O_19_; CAS: 142809-89-0; MW: 959.12) on the viability of bladder cand colon cancer cells, which interested us as we found that there was not yet any biological function assigned to this natural compound; there are only few publications on the detection or isolation of clinopodiside A.

In the present study, we investigated the anticancer effect of clinopodiside A alone or in combination with a clinical drug *in vitro* and *in vivo*, as well as examined the underlying mechanisms. The study was designed under the perceptions that: 1) combination treatment is common in treating cancer; 2) assessment under *in vivo* conditions is an important step in drug discovery, as *in vitro* and *in vivo* effects can be different or even opposite (Zogovic et al., 2009); and 3) understanding of the mechanisms underlying the anti-cancer effect of a compound benefits its therapeutic development.

## 2 Materials and methods

### 2.1 Chemicals, reagents, cells, and antibodies

Clinopodiside A was purchased from Chengdu DeSiTe Biological Technology Co., Ltd, Chengdu, China. The purity of clinopodiside A reached 96.9%, which was dissolved in dimethyl sulfoxide (DMSO) and kept at 4°C till use. 5-fluorouracil, cisplatin and methyl thiazolyl tetrazolium (MTT) were purchased from Sigma-Aldrich (St. Louis, MO). The mouse antibodies against Gasdermin D, LC3B-I, LC3B-II, DOCK4, BLK3, ARHGEF6, RHOU, RasGRP2, and GAPDH as well as the secondary antibody (goat anti-mouse) were purchased from Sangon Biotech (Shanghai, China). The T24 bladder and HCT116 cancer cells were obtained from the National Collection of Authenticated Cell Cultures (Shanghai, China).

### 2.2 Cell culture and treatment

The cancer cells were cultured in DMEM medium supplemented with 10% fetal bovine serum and antibiotics under 37°C in a humidified incubator with 5% CO_2_. All of the experiments were performed while the cells density reached approximately 70%. Chemical was added directly into cell culture to the desired working concentration in the *in vitro* experiments. The samples treated for 48 h were used for cell viability assay, while other samples were collected at different time points for the western blot analysis. The bladder cancer cells treated for 10 h with clinopodiside A were used for transcriptomic profiling. In the control group, cells were treated with equal amount of the solvent. 3-methyladenine (3-MA) was used to pre-treat the cells for 3 h before the experimental treatment by clinopodiside A, which aimed to inhibit autophagy or the cytotoxicity of the natural product.

### 2.3 Cell viability assay

The cancer cells were seeded into 96-well plate (6,000 cells per well) and cultured for 24 h before treatment for 48 h, after which 10 µl MTT reagent (5 mg/ml) was added to each well and incubated for 4 h at 37°C in an incubator. Then, the medium was removed, and 150 µl DMSO was added to dissolve the crystals (gentle shaking at room temperature for 10–15 min). The absorbance at 490 nm was recorded on a spectrophotometer, which represents the level of cell viability which infers the cell growth/proliferation.

### 2.4 Cancer cell xenograft model

Institutional approval was obtained for the experimental protocol. Nude mice (BALB/c nu/nu mice, 6 weeks old) were purchased from the Institute of Laboratory Animals Science, Beijing, China. The cancer cells (1×10^7^ cells/ml, 100 µl) were injected subcutaneously into the left forelimbs of each mouse. Mice were randomly assigned into different experimental groups: 1) control; 2) clinopodiside A; 3) cisplatin; and 4) combinational treatment with clinopodiside A and a clinical drug. The compounds were given i.p., at the doses of: clinopodiside A (25 and 50 mg/Kg/d), cisplatin (2.5 mg/Kg/d) for 2 weeks. By the end of the treatment, the animals were sacrificed after anesthesia, the size of each dissected tumor tissues was measured. During the experiments, the body weights of mice were recorded at given time interval. Institutional approval was obtained for the experimental protocol.

### 2.5 Apoptosis analysis using V-FITC/PI staining and flow cytometry

The sub-G1 cells were quantified by flow cytometry after staining the control and treated bladder cancer cells using an annexin V-FITC/PI apoptosis detection kit (Vazyme Biotechnology, Nanjing, China). The cells were collected at a concentration of 1×10^5^ cells/ml, mixed with annexin V-FITC and PI, according to the protocol provided by manufacturer, and analyzed using a flow cytometer (ACEA Bioscience, San Diego, CA, United States).

### 2.6 Quantification of protein level using Western blot analysis

The cells were washed twice with cold PBS, lysed with ice-cold lysis buffer (25 mM Tris·HCl, pH 7.4, 150 mM NaCl, 1 mM EDTA, 1% NP-40, and 5% glycerol) with protease inhibitors cocktail (Beyotime Biotechnology, Shanghai, China). The lysates were centrifuged at 4°C for 30 min at 13,000 rpm, and the supernatants were collected. Protein concentrations of the lysates were determined using BCA protein assay kit (Beyotime Biotechnology, Shanghai, China). The cellular proteins were separated by polyacrylamide gel electrophoresis, blotted onto PVDF membrane which was then blocked with non-fat milk, washed, incubated with primary antibody, washed, incubated with secondary antibody, and washed. The images of protein bands were captured using chemiluminescence Imaging System (Clinx, Shanghai, China) and analyzed using ImageJ software.

### 2.7 Visualization of autophagosomes using MDC staining

The bladder cancer cells were cultured on the glass coverslips for 24 h in 6-well plate, followed by the experimental treatment for 4 or 8 h. The autophagosomes were visualized using MDC fluorescent staining kit (Beyotime, Shanghai, China). In brief, when the treatment was completed, the adherent cells were washed with PBS for twice, incubated with 1 ml MDC staining solution in 37°C for 30 mines. Then, the MDC staining solution was removed, and the cells were washed with assay buffer for 3 times, and the slides were mounted with mounting solution containing DAPI. The images were captured under a fluorescence microscope, which were displayed on the computer and analyzed using ImageJ software package.

### 2.8 Transcriptomic profiling

Total RNA was isolated using the Trizol Reagent (Invitrogen Life Technologies, Carlsbad, United States), after which the concentration, quality and integrity of RNA were determined using a NanoDrop spectrophotometer (Thermo Scientific, CA, United States). Sequencing libraries were then generated according to the following steps: 1) mRNA was purified from total RNA using poly-T oligo-attached magnetic beads. Fragmentation was carried out using divalent cations under elevated temperature in an Illumina proprietary fragmentation buffer; 2) the first strand of cDNA was synthesized using random oligonucleotides and Super Script II, and the second strand cDNA synthesis was subsequently performed using DNA Polymerase I and RNase H; 3) the cDNA fragments of 400–500 bp in length were selected and purified using the AMPure XP system (Beckman Coulter, Beverly, CA, United States), which then were enriched using Illumina PCR Primer Cocktail in a 15 cycle PCR reaction. Products were purified and quantified using the Agilent high sensitivity DNA assay on a Bioanalyzer 2100 system (Agilent) to construct a sequencing library; and 4) the library was then sequenced on NovaSeq 6000 platform (Illumina, San Diego, United States) by Shanghai Personal Biotechnology Cp., Ltd (Shanghai, China). The transcriptome dates were analyzed as the following steps: 1) Mapper reading. The reference genome and gene annotation files were downloaded from genome website. Then the filtered reads were mapping to the reference genome using HISAT2 v2.0.5 software package; 2) Differential expression analysis. The difference expression of genes was analyzed by DESeq (1.30.0) with screened conditions as follows: expression difference multiple |log2FoldChange| > 1, significant *p*-value < 0.05. At the same time, *R* language Pheatmap (1.0.8) software package was used to perform bi-directional clustering analysis of all different genes of samples; 3) GO enrichment analysis. Using Gene Ontology database and topGO *R* language package to perform GO enrichment analysis on the differential genes, calculate *p*-value by hypergeometric distribution method (the standard of significant enrichment is *p*-value <0.05), and find the GO term with significantly enriched differential genes to determine the main biological functions performed by differential genes; and 4) KEGG enrichment analysis. Cluster Profiler (3.4.4) software was used to carry out the enrichment analysis of the KEGG pathway of differential genes, focusing on the significant enrichment pathway with *p*-value <0.05.

### 2.9 Overexpression of BLK

The entire coding region of BLK was obtained *via* the full sequence synthesis by Sangon Biotech company (Sangon, Shanghai, China), which was subcloned into the pCDNA3.1 plasmid vector using the *KpnΙ/BamHI* restriction enzymes (Supplementary Figure S1). The constructed plasmid pCDNA-BLK was transformed into *E. coli* DH5α for amplification, which was then sequenced by Beijing Genomics institution (BGI, Beijing, China) to confirm the correct cloning. The sequencing results were provided in the Supplementary Table S1. pCDNA-BLK plasmids were transfected into T24 cells using Lipo 2000 (Thermo Fisher Scientific, MA, United States). Lipo2000 or the plasmids alone was firstly incubated in DMEM without FBS for 5 min, then, Lipo2000 and plasmids were mixed and incubated for 20 min. Finally, the mixture of Lipo200 and the plasmids was added to the cell culture for treatment for 36 h. At the end of the transfection, the cells were lysed, and the total proteins were extracted for quantification of the protein level of BLK using Western blot analysis described in Section 2.6.

### 2.10 Knockdown of protein level

The shRNA sequences targeted to RHOU and RasGRP2 were also obtained *via* synthesis by Sangon Biotech company (Sangon, Shanghai, China). The shRNA sequences used for RHOU and RasGRP2 knockdown were provide in the Supplementary Table S2. The sense and antisense sequences of shRNA were annealed to form a complementary double sequence with two cohesive ends, which then were subcloned into the pSilencer2.1-U6 plasmid vectors using *BamHI/EcoRI* restriction enzymes (Supplementary Figure S2). They plasmids were amplificated and sequenced to confirm the correct construction of the plasmids. For the gene knockdown, these two plasmids were separately transfected into the bladder cancer cells, and the successful knockdown of the target proteins was confirmed using Western blot analysis.

### 2.11 Determination of combination index CI_50_

The combinational effect of clinopodiside A and cisplatin was analyzed using the Loewe Additivity Mathematical Modeling. The combination index (CI_50_) was obtained to determine the type of the combination effect: 1) CI_50_ < 1 evidences a synergism; 2) CI_50_ = 1 reflects an additive effect; and 3) CI_50_ > 1 indicates an antagonism. CI_50_ is calculated from the equation: CI_50_ = a/IC_50_ of clinopodiside A + b/IC_50_ of cisplatin. In the equation, IC_50_ values of clinopodiside A and cisplatin when used alone in the MTT assay, that showed 50% inhibition of the cancer cell viability; “a” was the IC_50_ of clinopodiside A when used together with cisplatin in the MTT assay; and “b” was the IC_50_ of cisplatin when used together with clinopodiside A in the cell viability assay.

### 2.12 Statistical analysis

The experiments were performed in triplicate. All data were represented as mean ± SD. Statistical analysis was performed using SPSS computer analysis software package. Student’s-*t* test was used for the comparison between two groups. Multi-group comparisons were performed using one way ANOVA. A *p* < 0.05 was considered to be statistically significant. *: *p* < 0.05, **: *p* < 0.01, and ***: *p* < 0.001, compared with the control group. ^#^: *p* < 0.05 and ^##^: *p* < 0.01, compared with the treated group.

## 3 Results

### 3.1 Clinopodiside A alone or in combination with a clinical drug inhibits the viability of the bladder and colon cancer cells *in vitro*

**FIGURE 1:**
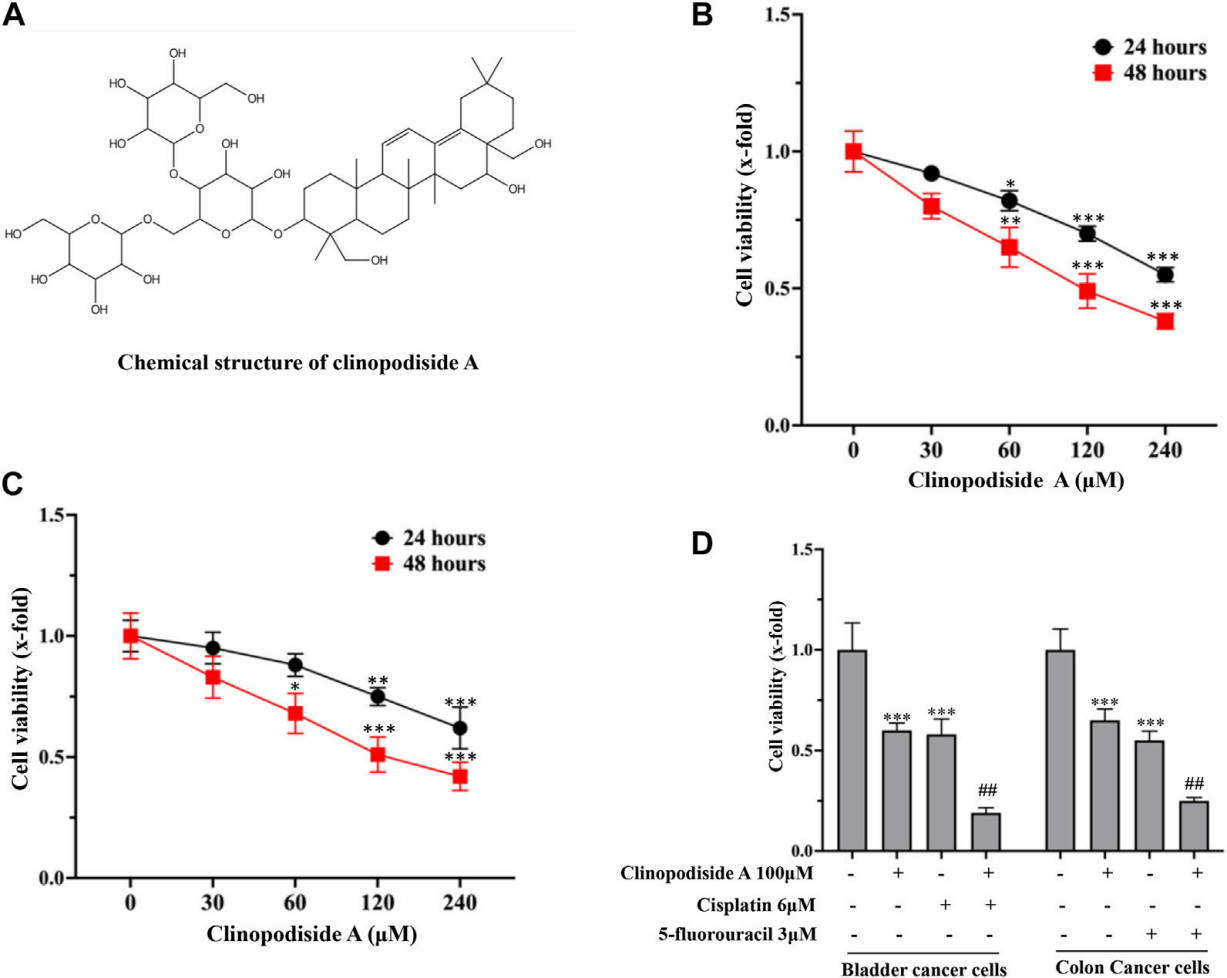
Clinopodiside A *in vitro* inhibits the growth of the T24 bladder and HCT116 colon cancer cells in concentration- and time-dependent manners, which showed the combinational effects when used with cisplatin or 5-fluorouracil. **(A)** The chemical structure of clinopodiside **(A–C)** Clinopodiside A inhibited the proliferation of the bladder **(B)** and colon **(C)** cancer cells in concentration- and time-dependent manners. *X*-axis was the concentration of clinopodiside A used for the treatments. *Y*-axis was the cell viability (x-fold). The *p*-values were obtained by comparing with the controls **(D)** clinopodiside A showed combinational effects when used with cisplatin or 5-fluorouracil for the bladder and colon cancer cells, respectively. MTT assay was used to quantify the level of cell viability, which indicates the level of cell growth/proliferation. *: *p* < 0.05; **: *p* < 0.01; ***: *p* < 0.001, compared with the control group; ##: *p* < 0.01, compared with the groups treated with a compound alone. We performed each experiment at least in triplicate.

Figure 1A showed the chemical structure of clinopodiside A. When applied to the cell culture, it was able to inhibit the viability of the T24 bladder and HCT116 colon cancer cells (Figures 1B,C, respectively) in a concentration- and time-dependent manner. The compound showed a combinational effect in the inhibition of the cancer cell viability, when used together with cisplatin or 5- fluorouracil (Figure 1D) which are frontline chemotherapeutic drugs for the treatments of bladder and colon cancers, respectively. This indicates that clinopodiside A can inhibit the growth of the bladder and colon cancer cells *in vitro*, when used alone and in combination with cisplatin or 5-fluorouracil.

### 3.2 Clinopodiside A alone or in combination with clinical drug inhibits the growth of the bladder and colon cancer cells *in vivo*

**FIGURE 2:**
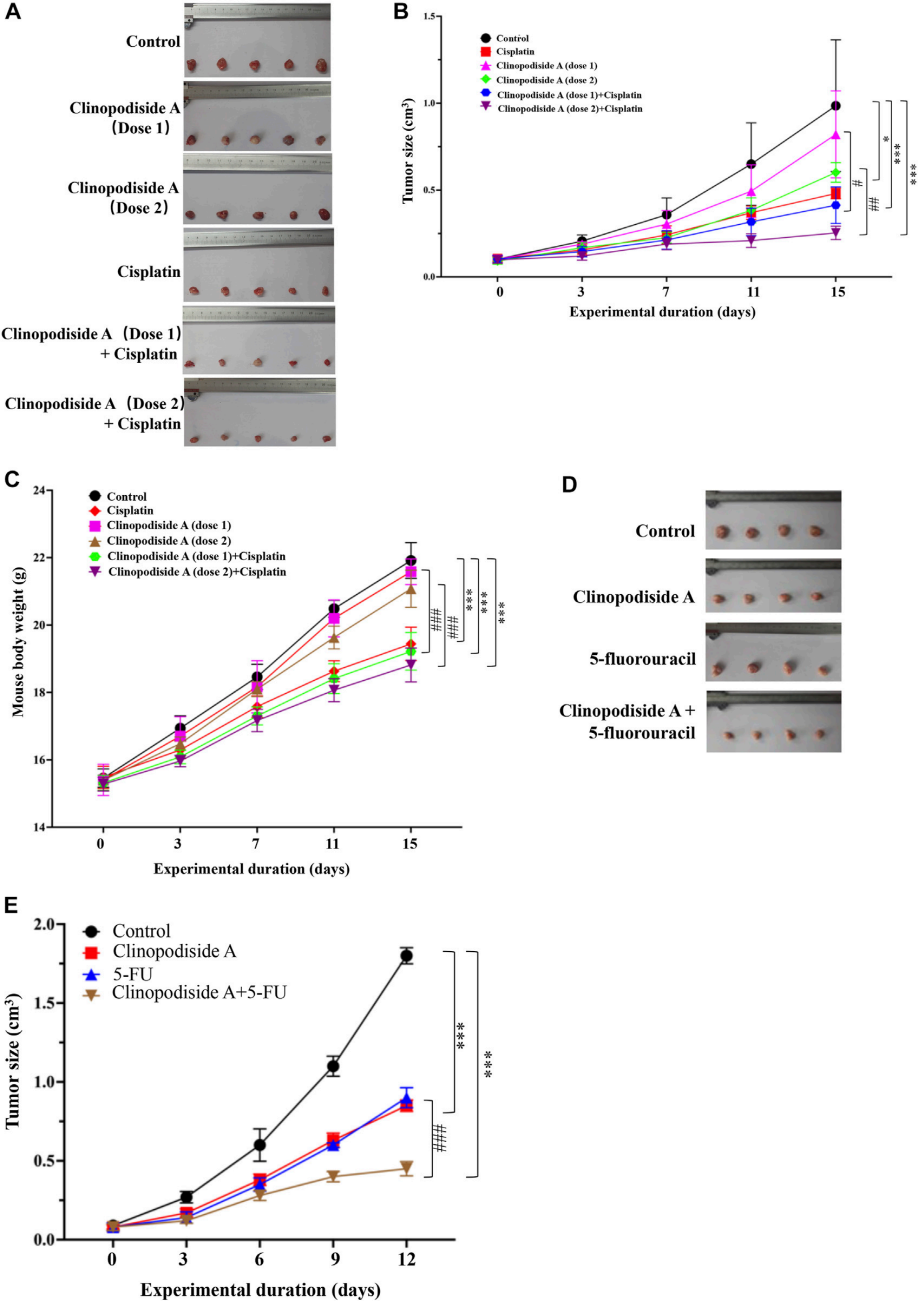
Clinopodiside A *in vivo* inhibits the growth of the T24 bladder and HCT116 colon cancer cells, which showed the combinational effects when used with cisplatin or 5-fluorouracil. **(A,B)** Clinopodiside A inhibited the growth of the bladder cancer cells in a dose-dependent manner in the nude mice. There was a trend of a combinational effect with cisplatin. The *p*-values were obtained by comparing with control group. The results from experimental groups at day 15 were used for the comparisons; **(C)** The body weight of the mice. Cisplatin reduced the body weight of the mice substantially (*p* < 0.001), while clinopodiside A only slightly reduced the body weight of the mice (*p* < 0.05), when used at high dose. **(D,E)** Clinopodiside A inhibited the growth of the colon cancer cells in a dose-dependent manner in the nude mice. There was a combinational effect with 5-fluorouracil. Clinopodiside **(A)** dose 1 = 25 mg/kg/d, dose 2 = 50 mg/kg/d; Cisplatin: 2.5 mg/kg/d. 5-FU = 12 mg/kg/d *: *p* < 0.05; ***: *p* < 0.01, compared with the control group. ###: *p* < 0.001, compared with the groups treated with a compound alone. The control for **(A–E)**: solvent for clinopodiside A, which is DMSO. We performed each experiment at least in triplicate.

Clinopodiside A was able to inhibit the growth of the bladder cancer cells in a dose-dependent manner in the nude mice, which showed combinational effects when used together with cisplatin (Figures 2A,B). Cisplatin substantially reduced the body weight of the mice during the treatment period (*p* ≤ 0.001) (Figure 2C). In contrast, clinopodiside A did not reduce the body weight of the mice, when was used at the dose of 25 mg/kg/d. It only slightly lowered the mice body weight when used at the dose of 50 mg/kg/d (Figure 2C). This indicates that clinopodiside A is much better tolerated than the synthetic drug cisplatin by the mice. In order to reveal the combinational effect, we did not used the compounds at the doses that could inhibit the growth of the cancer cells by 60% or over. Similarly, clinopodiside A inhibited the growth of HCT116 colon cancer cells in the nude mice, which showed a combinational effect when used with 5-fuorouracil (Figures 2D,E).

### 3.3 Clinopodiside A inhibits the viability of the bladder cancer cells *via* autophagy

**FIGURE 3:**
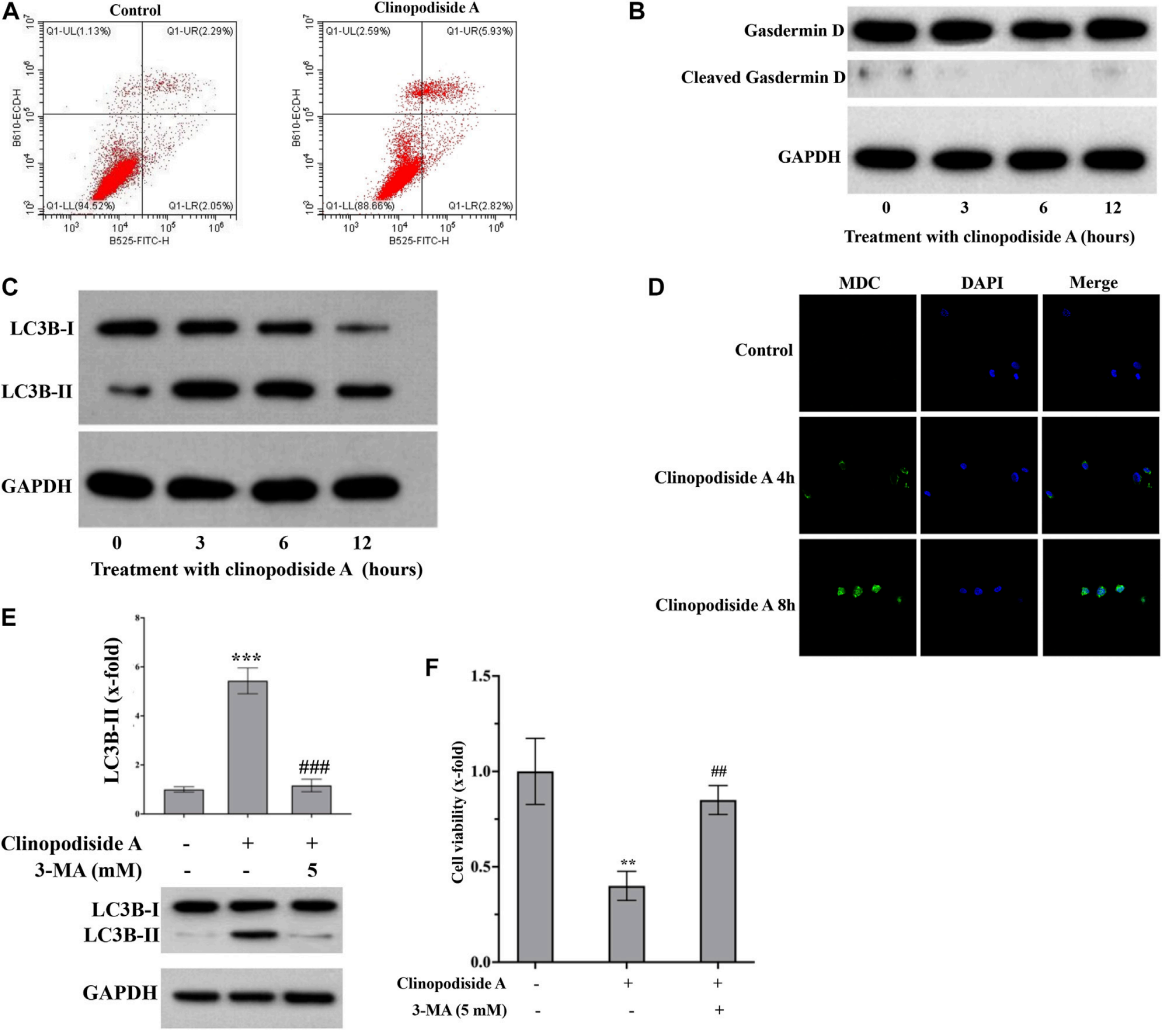
Clinopodiside A inhibits the proliferation of the bladder cancer cells *via* autophagy. **(A)** The results from the flow cytometry analysis, which showed the evidence that clinopodiside A did not cause necrosis or apoptosis of the cancer cells. **(B)** The Western blot analysis for the cleavage of Gasdermin **(D)**. Clinopodiside A did not induce the cleavage of Gasdermin D, which suggests that the compound does not cause pyroptosis, as the cleavage of Gasdermin D is typical evidence for pyroptosis. **(C)** Western blot analysis for the LC3B-II, which is typical evidence for autophagy. **(D)** The clinopodiside A-caused autophagy was further evidenced by the fluorescent staining of the autophagosome. **(E)** 3-MA which is recognized inhibitor of autophagy was able to inhibit the clinopodiside A-evoked LC3B-II. **(F)** 3-MA was able to attenuate the clinopodiside A-induced growth inhibition of the cancer cells. Clinopodiside **(A)** 100 μM; ***: *p* < 0.001, compared with the controls; ##: *p* < 0.01, compared with the treated group. The control for **(A,D)**: solvent for clinopodiside A, which is DMSO. We performed each experiment at least in triplicate. Amongst the panels of the flow cytometry analysis: UL = upper left; UR = upper right; LL = lower left; LR = lower right.

Next, we investigated whether and how clinopodiside A inhibited the cancer cell viability *via* inducing cell death. Annexin V-FITC/PI staining was performed followed by flow cytometry analysis of the control and treated cells to test the possibility of apoptosis. No obvious evidence for necrosis or apoptosis after the treatment by clinopodiside A, as the percentages of the cells in the panels Q1-UL, Q1-UR, and Q1-LR were not substantially altered by the experimental treatment (Figure 3A). Then, the cleavage of Gasdermin D was used to test whether the compound caused pyroptosis. Also, there was no evidence for pyroptosis, as the treatment did not cause the cleavage of Gasdermin D (Figure 3B). Interestingly, the clinopodiside A showed ability to elicit the LC3B-II (Figure 3C), a marker for autophagy. To further support the treatment-evoked autophagy, we attempted to visualize and compare the level of the autophagosome using MDC staining in the control and treated samples. Indeed, the clinopodiside A produced the autophagosome (Figure 3D). Moreover, the commonly used autophagy inhibitor 3-MA was not only able to inhibit the treatment-evoked LC3B-II (Figure 3E), but also could attenuate the compound-induced inhibition of the cell viability (Figure 3F). Thus, it is likely that clinopodiside A inhibits the viability of the bladder cancer cells *via* the induction of autophagy.

### 3.4 Transcriptomic profiling of clinopodiside A-evoked differential gene expressions in the bladder cancer cells

**FIGURE 4:**
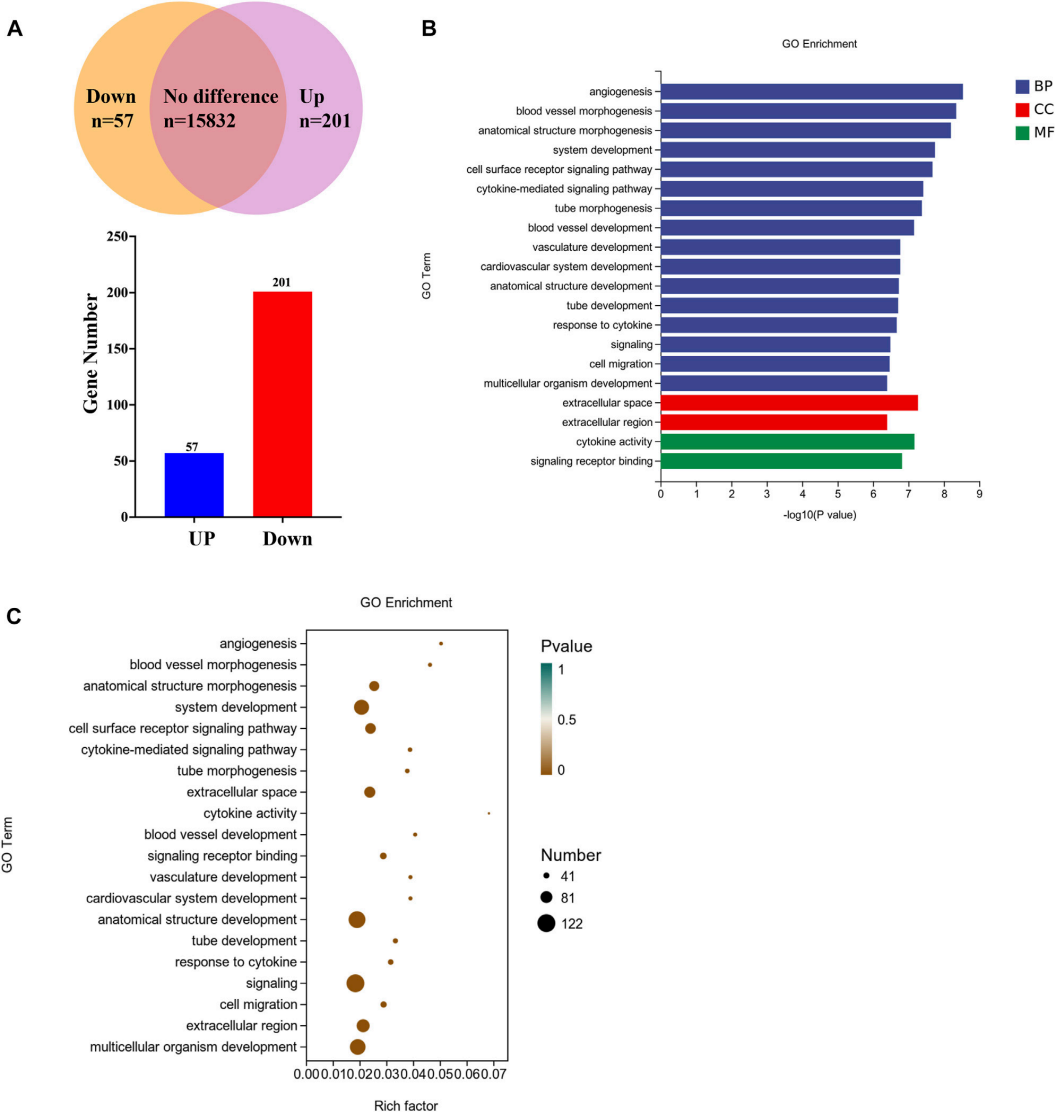
Transcriptomic profiling of the clinopodiside A-induced differential gene expressions. **(A)** Summary of the number of mRNA species detected, up-regulated or downregulated. **(B)** GO annotation of the differentially expressed genes, which showed that they were mostly clustered in the category of biological process. **(C)** Deepened analysis of the GO terms, which showed that some genes were enriched in the terms of “signaling” and “signaling receptor binding”. This provided directions for us to choose candidates for further function analysis, which included RasGRP2 and RHOU from the term of “signaling” (GO ID: 0023052; number 18 in the enrichment list; Supplementary Table S3) and BLK and DOCK4 from the term of “signaling receptor binding” (GO ID: 0005102, number 12 in the enrichment list; Supplementary Table S3). ARHGEF16 which was enriched in both terms was also included. We performed each experiment at least in triplicate.

To investigate the molecular basis of the autophagy, firstly transcriptome analysis was performed to identify clinopodiside A-caused differential gene expressions. A total of 16,090 mRNA species were identified, amongst which the expression levels of 258 genes were altered by the experimental treatment (Figure 4A); specifically, 57 and 201 genes were up-regulated and down regulated, respectively. Bioinformatic annotation using Go enrichment revealed that the 258 differentially expressed genes were mostly enriched in the category of biological process, while some enriched in the cellular component and molecular function categories (Figure 4B). Further annotation was performed in an attempt to retrieve meaningful information from the terms. Data revealed that genes in the “signaling” and “signaling receptor binding” terms were significantly enriched (Figure 4C). Therefor the RasGRP2 and RHOU were selected from the term of signaling (GO ID: 0023052; Supplementary Table S3) as well as BLK and DOCK4 from the term of signaling receptor binding (GO ID: 0005102; Supplementary Table S3) for further functional analysis. ARHGEF16 which was enriched in both terms was also included.

### 3.5 Clinopodiside A induces the autophagy *via* the signaling of BLK and RasGRP2

**FIGURE 5:**
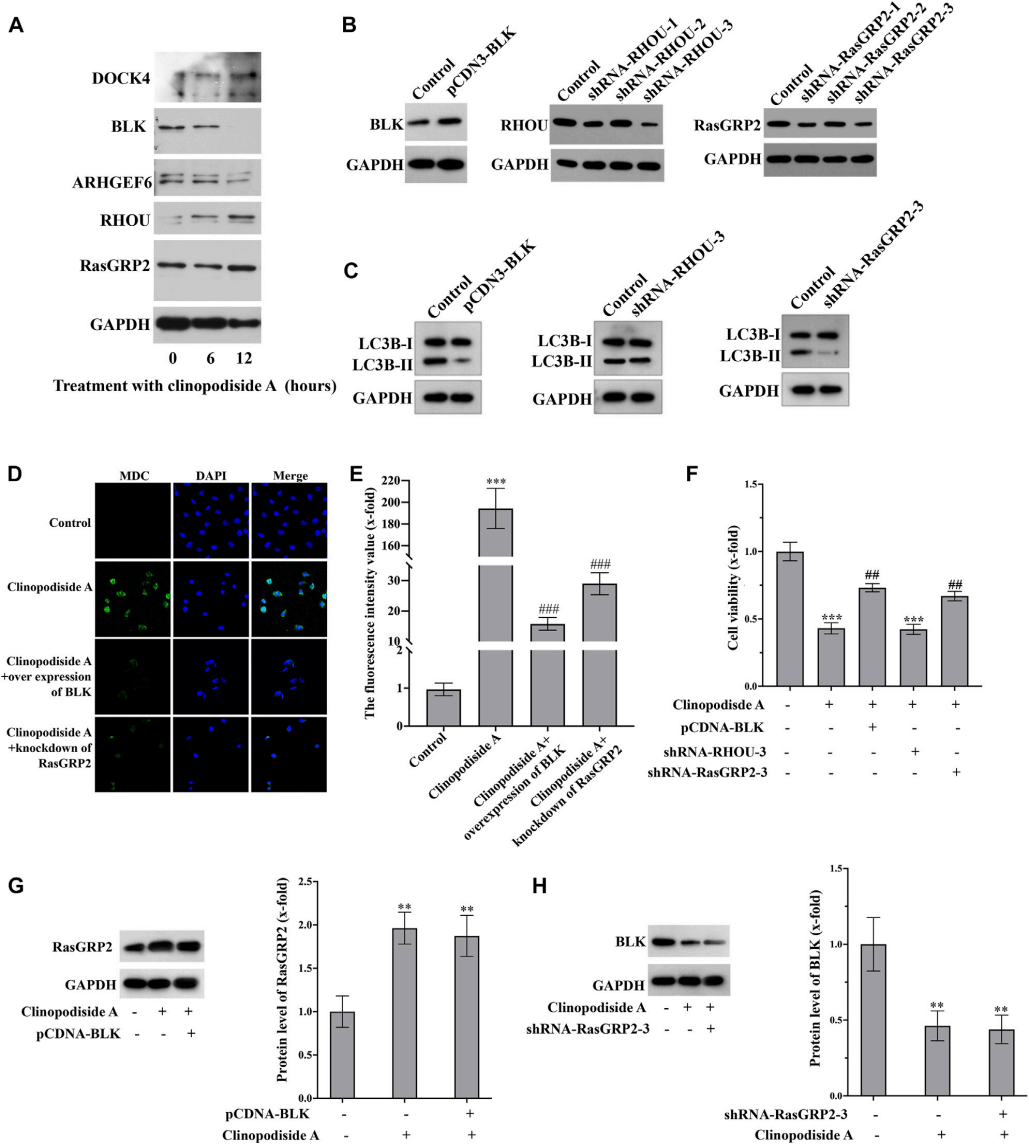
Clinopodiside A causes the autophagy *via* the signaling of BLK and RASGRP 2. **(A)** Western blot analysis showed that clinopodiside A decreased the protein level of BLK, but increased the expression of RasGRP2, RHOU, and DOCK4. The protein level of ARHGEF6 remained unchanged. **(B)** Western blot analysis confirmed the successful over-expression of BLK using pCDNA3.1 plasmid vector and the knockdown of the protein levels of RasGRP2 and RHOU using their specific shRNA species. **(C)** Over-expression of BLK or knockdown of RasGRP2, but not knockdown of RHOU, inhibited the LC3B-II. **(D)** Either BLK overexpression or RasGRP2 knockdown could inhibit clinopodiside A-caused autophagy revealed by the MDC staining. **(E)** The normalized fluorescence intensities of the pictures in **(D)**. **(F)** Over-expression of BLK or knockdown of RasGRP2 attenuated the growth inhibition effect of clinopodiside **(A,G)** Over-expression of BLK did not modify the expression of RasGRP2. **(H)** Knockdown of RasGRP2 did not alter the expression of BLK. The controls for **(B,C)**: the vector for protein overexpression or siRNA, as applicable; The control for **(D)**: solvent for clinopodiside A, which is DMSO. The concentration of clinopodiside A used: 120 µM. We performed each experiment at least in triplicate.

The Western blot analysis (Figure 5A) showed that the experimental treatment increased the protein levels of RasGRP2 and RHOU, while decreased the expression of BLK. The protein level of ARHGEF16 remain unchanged. The antibody against DOCK4 produced multiple bands, which was excluded for further analysis, as we were unsure whether the treatment did increase its expression. Functionally, over-expression of BLK (Figure 5B) inhibited the treatment-evoked LC3B-II (Figure 5C) and the autophagy revealed by the MDC staining (Figures 5D,E), which also attenuated the cytotoxicity of clinopodiside A (Figure 5F). Knockdown of RasGRP2 (Figure 5B) also inhibited the LC3B-II (Figure 5C), the autophagy by the MDC staining (Figure 5D), and the cytotoxicity of clinopodiside A (Figure 5F). Knockdown of RHOU (Figure 5B) neither modify the conversion of LC3 (Figure 5C), nor changed the cytotoxicity of the compound (Figure 5F). The assessment of the relationship between BLK and RasGRP2 was clarified that in the clinopodiside A-treated cells, over-expression of BLK did not change the protein level of RasGRP2 (Figure 5G), and knockdown of RasGRP2 had no effect on the expression of BLK (Figure 5H). Therefore, BLK and RasGRP2 are likely to represent two independent signaling pathways for the compound-induced autophagy.

### 3.6 Clinopodiside A and cisplatin have a synergistic inhibition effect on the growth of the bladder cancer cells

**FIGURE 6:**
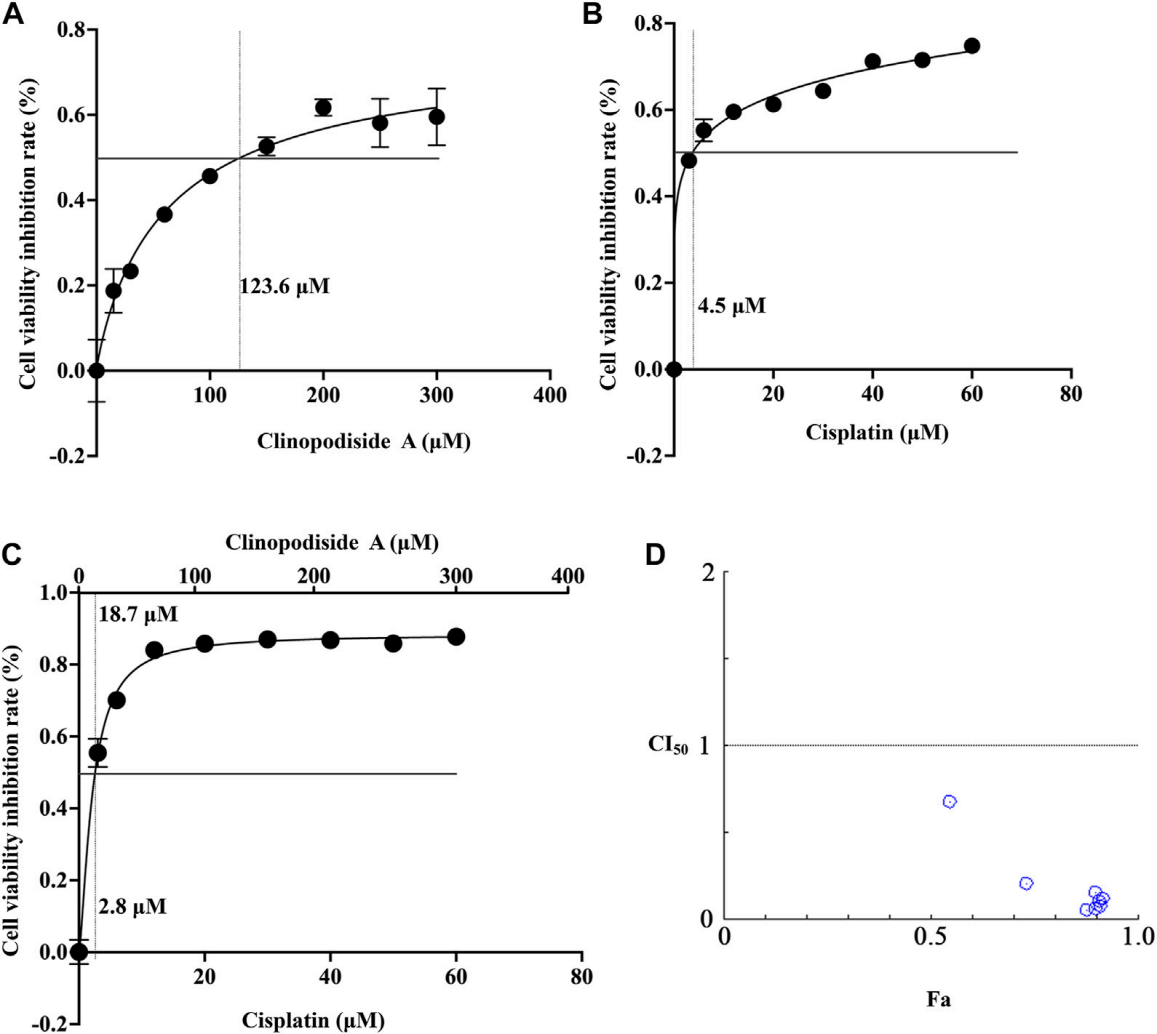
Examination of the combinational effect between clinopodiside A and cisplatin using mathematical modeling. **(A)** The fitting curve of clinopodiside A concentration and cell viability inhibition rate, when used alone. The IC_50_ value of clinopodiside A was 123.6 μM. **(B)** The fitting curve of cisplatin concentration and cell viability inhibition rate, when used alone. The IC_50_ of cisplatin was 4.5 μM. **(C)** The fitting curve of clinopodiside A and cisplatin concentrations by plotting against cell viability inhibition rate, when clinopodiside A and cisplatin were used in combination. The IC_50_ values of clinopodiside A and cisplatin were 18.7 and 2.8 μM, respectively. **(D)** The combination indexes (CI_50_ values) when clinopodiside A and cisplatin were used at different concentrations. We performed each experiment at least in triplicate.

The mechanism underlying the combinational effect between clinopodiside A and cisplatin was elucidated in (Figures 1D, 2A,B). The IC_50_ values for clinopodiside A and cisplatin, as shown in Figures 6A,B, were 123.6 and 4.5 μM, respectively, when the compounds were used alone. When clinopodiside A and cisplatin were used in combination, the “a” and “b” were 18.7 and 2.8 μM, respectively (Figure 6C). Upon this, the CI_50_ value was 0.77, which was less than 1, suggesting a synergistic effect between clinopodiside A and cisplatin. The CI_50_ values appeared to decrease along the increase in the doses of the clinopodiside A and cisplatin, all of which were less than 1 (Figure 6D). The synergism of clinopodiside A and cisplatin for inhibition of the bladder cancer cell viability was seen in the dose ranges of 15–300 μM for clinopodiside A and 3–60 μM for cisplatin.

### 3.7 The combinational use of clinopodiside A and cisplatin augments the autophagy and cisplatin-triggered apoptosis

**FIGURE 7:**
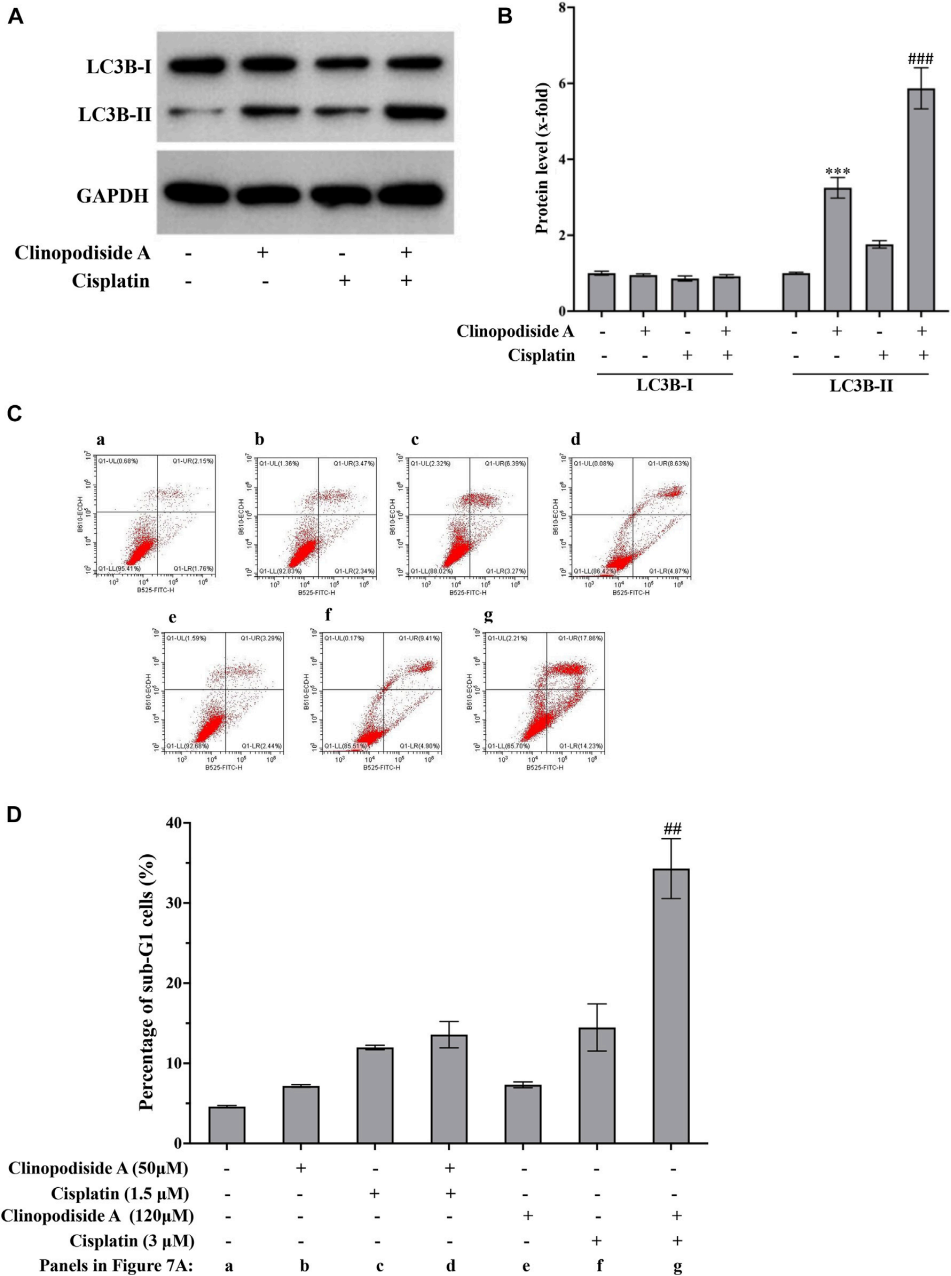
The combinational treatment using clinopodiside A and cisplatin increased the level of autophagy and the cisplatin-triggered apoptosis in the bladder cancer cells. **(A,B)** Western blot analysis showed that cisplatin was also able to induce autophagy, as evidenced by the increasing of LC3B-II. The combinational use of the clinopodiside A and cisplatin resulted in an increased level for LC3B-II. Clinopodiside **(A)** 50 μM; cisplatin: 4 μM. **(C,D)** Cisplatin, but not clinopodiside A (Figure 3A), could induce apoptosis of the bladder cancer cells. The combinational use clinopodiside A and cisplatin resulted in an increased level apoptosis, suggesting that clinopodiside A promotes the cisplatin-elicited apoptosis. The treatment time for samples for the Western blot analysis: 8 h; The treatment time for the samples for flow cytometry analysis: 36 h ##: *p* < 0.01, compared with groups treated with one compound. We performed each experiment at least in triplicate.

The mechanisms of the combinational effect between clinopodiside A and cisplatin were investigated by examined the autophagy and apoptosis in different samples. The results showed that cisplatin itself could cause both autophagy and apoptosis (Figures 7A–D). The combinational treatment increased the autophagy, as evidenced by the level of LC3B-II (Figures 7A,B). Interestingly, clinopodiside A was able to enhance the cisplatin-evoked apoptosis, as evidence by that the apoptosis level was higher in the combinational treatment group than that in the sample treated by cisplatin alone (Figures 7C,D). Thus, it appears that the combinational effect of the two compounds is attributed to increased levels of autophagy and the cisplatin-elicited apoptosis, and the autophagy promotes the apoptosis.

## 4 Discussion

Our results showed that clinopodiside A was able to inhibit the growth of the bladder cancer cells, which, to our knowledge, is the first biological function assigned to this natural compound. The *in vitro* IC_50_ values for cisplatin and clinopodiside A were 4.5 and 123.6 μM respectively, for the bladder cancer cells, which shows that cisplatin is far more potent that clinopodiside A in the inhibition of the cancer cell proliferation. However, it is noticed that although cisplatin (2.5 mg/kg/d) and clinopodiside A (50 mg/kg/d) achieved comparable levels growth inhibition on the cancer cells *in vivo*, cisplatin significantly decreased the body weight of the nude mice within 2 weeks. In contrast, clinopodiside A only slightly reduced the mice body weight. Thus, it appears that clinopodiside A is much better tolerated by the animals, and its use is thus less likely to be restricted by dose-limiting effect. The natural compound can be used at effective doses without significant toxic side-effects.

In the study, the BLK and RasGRP2 were identified as signaling mediators for the autophagy, which implicates that BLK and RasGRP2 are new members of autophagy regulators, as we failed to retrieve any existing report that BLK or RasGRP2 plays a role in autophagy. BLK expression level can be controlled by c-myc (Zhang et al., 2012a) which can regulate autophagy (Hwang et al., 2020). Similarly, RasGRP2 can signal *via* PI3k-AKT pathway which mediates autophagy *via* mTOR (Xu et al., 2020). The indirect evidence supports that BLK and RasGRP2 may indeed players in the regulation of autophagy. Moreover, we did not find any published report that BLK and RasGRP2 have a functional relationship. In agreement with this, our results showed that BLK and RasGRP2 mediated the clinopodiside A-evoked autophagy independently, without being in direct upstream or downstream relationship. Nevertheless, it is unknown whether BLK and RasGRP2 could share upstream or downstream signaling molecules, or they can bind to each other.

BLK is a Src family of non-receptor tyrosine kinase, which is encoded on chromosome 8p23.1 that is mainly expressed in B lymphoid cells to regulate the proliferation and differentiation. According to Tretter et al. (2003), BLK plays an important role during B lymphoid cell ontogeny by contributing to the assembly of the pre-B cell receptor, a critical checkpoint in the transition of B lymphoid cells from pro-B cells to pre-B cells. Furthermore, BLK regulates the immune tolerance of B lymphocytes (Hom et al., 2008). In relevance to cancer, BLK displays opposing roles; it is considered to be a proto-oncogene that increases tumor cell proliferation (Borowiec et al., 2009). In contrast, there is evidence that BLK can inhibit tumor progression, i.e., in a mouse model, it inhibits the progress of chronic myeloid leukemia *via* increasing the expression of p27 (Zhang et al., 2012b). In the present research, clinopodiside A promoted autophagy in the bladder cancer cells by reducing BLK protein level, which supports the view that BLK favors cancer cell survival. It appears that the exact role of BLK in cancer development depends on different situations such as the type of cancer.

RasGRP2 is encoded by a gene located on chromosome 11q13.1, which is a calcium and diacylglycerol-regulated guanine nucleotide exchange factor that specifically activates Rap through the exchange of bound GDP for GTP. RasGRP2 typically regulates platelet function *via* inducing Rap1 activation that is essential to trigger fibrinogen binding to the αIIbβ3 integrin, which leads to the thrombi formation to stop bleeding (Eto et al., 2002; Crittenden et al., 2004). Furthermore, RasGRP2 is found to regulate the development and maturation progress of immune cell; in megakaryocyte, RasGRP2 expression *via* the transcription factor NE-F2 promotes the differentiation of megakaryocytic at late phase (Zang et al., 2016). In relevance to the cell fate, it can act as a proto-oncogene, promoting cell proliferation and inhibiting apoptosis (Dupuy et al., 2001). In the present study, we discovered that clinopodiside A promoted RasGRP2 expression and caused autophagy in the bladder cancer cells, which is in contrast to the reports that RasGRP2 favors cell death. Since Rap1 is the most common target of RasGRP2, which is a critical coordinator of the lysosomal system (Mutvei et al., 2020) and dysregulation of lysosomal system and is associated with autophagy (Zhang et al., 2021). We suspect that RasGRP2 regulated autophagy in the bladder cancer cells *via* Rap1-lysosomes pathway.

Apoptosis and autophagy are both well controlled biological events that play crucial role in body development, metabolic homeostasis and diseases. Apoptosis appears to be the most-studied form of programmed cell death (Green, 2005), whereas autophagy is usually recognized as a response to the stress to avoid cell death and an important alternative cell death pathway (Levine and Kroemer, 2019). Interestingly, there are molecular and functional intertwines between apoptosis and autophagy, which can be summarized into the following manners: 1) Antagonistic effect. In many cases, apoptosis is the consequence of the failure of autophagy to resistance to the stress; in mouse model, the inhibition of autophagy by knockout Atg5 results in cell apoptosis in neural cells (Hara et al., 2006). Conversely, apoptosis can have an inhibitor effect on autophagy (Lum et al., 2005). At the molecular level, Beclin1, a critical protein for autophagy, has been found to be a substrate of Caspase 3. The cleaved Beclin1 loses its ability to trigger autophagy, and the C-terminal fragment can localize to mitochondria to facilitate apoptosis (Wirawan et al., 2010). Promoting effect. LC3, a crucial component in autophagosome elongation and enclosure, can function as a platform for apoptosis by promoting caspase-8 activation; it interacts with SQSTM1/p62 and caspase-8 to form a complex, which leads to caspase-8 aggregation and autoactivation, eventually resulting in apoptosis (Young et al., 2012). On the other hand, there is a lack of evidence that apoptosis can promote autophagy; 2) Apoptosis and autophagy are triggered simultaneously by the stimuli such drugs. ROS is common signal that can induce both apoptosis *via* increasing the mitochondrial outer membrane permeabilization (Huai et al., 2013), and autophagy *via* stimulating the proteolytic activity of ATG4 (Scherz-Shouval et al., 2007). In our study, the combinational use of clinopodiside A and cisplatin enhanced the autophagy and apoptosis. It appears that the combinational use of the compounds increases the autophagy in an additive or synergistic manner, and that autophagy upregulates the apoptosis. At the molecular level, we don’t know whether BLK and RasGRP2 are involved in the crosstalk between the autophagy and apoptosis in our combinational treatment.

In conclusion, our results suggest that clinopodiside A inhibits the growth of the bladder cancer cells *via* the induction of autophagy which is mediated by BLK and RasGRP2. The synergism between clinopodiside A and cisplatin is due to the increased autophagy and the autophagy-promoted apoptosis. Clinopodiside A is a promising investigational drug for cancer chemotherapy, which can be used alone or in combination with a clinical drug.

## Data Availability

The datasets presented in this study can be found in online repositories. The names of the repository/repositories and accession number(s) can be found below: https://doi.org/10.6084/m9.figshare.20250264.
