# Supplementary material for: The novel antitumor compound clinopodiside A induces cytotoxicity via autophagy mediated by the signaling of BLK and RasGRP2 in T24 bladder cancer cells
Source: Front Pharmacol. 2022 Sep 19;13:982860. doi: 10.3389/fphar.2022.982860 (PMC9527273; doi:10.3389/fphar.2022.982860)
Supplement: Supplementary file 2 [file Table1.DOCX]

**Supplementary materials**

**Table 1. BLK full sequence.**

ATGGGGCTGGTAAGTAGCAAAAAGCCGGACAAGGAAAAGCCGATCAAAGAGAAGGACAAGGGCCAATGGAGCCCCCTGAAGGTCAGCGCCCAAGACAAGGACGCCCCGCCACTGCCGCCCCTGGTTGTCTTCAACCACCTTACTCCTCCACCGCCCGATGAACACCTGGATGAAGACAAGCATTTCGTGGTGGCTCTGTATGACTACACCGCTATGAATGATCGGGACCTGCAGATGCTGAAGGGGGAGAAGCTACAGGTCCTGAAGGGAACTGGAGACTGGTGGCTGGCCAGGTCACTCGTCACAGGAAGAGAAGGCTATGTGCCCAGTAACTTTGTGGCCCGAGTGGAGAGCCTGGAAATGGAAAGGTGGTTCTTTAGATCACAGGGTCGGAAGGAGGCTGAGAGGCAGCTTCTTGCTCCAATCAACAAGGCCGGCTCCTTTCTTATCAGAGAGAGTGAAACCAACAAAGGTGCCTTCTCCCTGTCTGTGAAGGATGTCACCACCCAGGGGGAGCTGATCAAGCACTATAAGATCCGCTGCCTGGATGAAGGGGGCTACTACATCTCCCCCCGGATCACCTTCCCCTCGCTCCAGGCCCTGGTGCAGCACTATTCTAAGAAGGGGGATGGTCTATGCCAGAGGCTGACCCTGCCCTGTGTGCGCCCGGCCCCGCAGAATCCCTGGGCCCAGGATGAATGGGAGATCCCCCGGCAGTCTCTCAGGCTGGTCAGGAAACTCGGGTCTGGACAATTCGGCGAAGTCTGGATGGGTTACTACAAAAACAACATGAAGGTGGCCATTAAGACGCTGAAGGAGGGAACCATGTCTCCAGAAGCCTTTCTGGGTGAGGCCAACGTGATGAAGGCTCTGCAGCACGAGCGGCTGGTCCGACTCTACGCAGTGGTCACCAAGGAGCCCATCTACATTGTCACCGAGTACATGGCCAGAGGATGCCTGCTGGATTTCCTGAAGACAGATGAAGGGAGCAGATTGTCACTCCCAAGGCTGATTGACATGTCGGCGCAGATTGCTGAAGGGATGGCATACATTGAGCGCATGAATTCCATCCACCGCGACCTGCGGGCGGCCAACATCCTGGTGTCTGAGGCCTTGTGCTGCAAAATTGCTGATTTTGGCTTGGCTCGAATCATCGACAGTGAATACACGGCCCAAGAGGGGGCCAAGTTCCCCATCAAGTGGACAGCCCCGGAAGCCATCCACTTCGGGGTCTTCACCATCAAAGCAGACGTGTGGTCGTTTGGAGTCCTCCTGATGGAAGTTGTCACTTATGGGCGGGTGCCATACCCAGGGATGAGCAACCCCGAGGTCATCCGCAACCTGGAGCGCGGCTACCGCATGCCGCGCCCCGACACCTGCCCGCCCGAGCTGTACCGCGGCGTCATCGCCGAGTGCTGGCGCAGCCGGCCCGAGGAGCGGCCCACCTTCGAGTTCCTGCAGTCGGTGCTGGAGGACTTCTACACGGCCACCGAGCGGCAGTACGAGCTGCAGCCCTAG

**Figure 1. The BLK overexpression plasmid map.**

**Table 2. The shRNA sequence.**

**ShR-RasGRP2 sequence:**

ShR-RasGRP2-1

TOP: 5’-GATCCAATCCCGGAAGGACAACTCCACTCGAGTGGAGTTGTCCTTCCGGGATTTTTTTGA-3’

BOT: 5’-AGCTTCAAAAAAATCCCGGAAGGACAACTCCACTCGAGTGGAGTTGTCCTTCCGGGATTG-3’

ShR-RasGRP2-2

TOP: 5’-GATCCAAGGAGCTGAAGGCTCTGCTACTCGAGTAGCAGAGCCTTCAGCTCCTTTTTTTGA-3’

BOT: 5’-AGCTTCAAAAAAAGGAGCTGAAGGCTCTGCTACTCGAGTAGCAGAGCCTTCAGCTCCTTG-3’

ShR-RasGRP2-3

TOP: 5’-GATCCCACAACTTCCAGGAGAGCAACCTCGAGGTTGCTCTCCTGGAAGTTGTGTTTTTGA-3’

BOT: 5’-AGCTTCAAAAACACAACTTCCAGGAGAGCAACCTCGAGGTTGCTCTCCTGGAAGTTGTGG-3’

**ShR-RHOU sequence:**

ShR-RHOU-1

TOP: 5’-GATCCCGGACAGGATGAATTTGACAACTCGAGTTGTCAAATTCATCCTGTCCGTTTTTGA-3’

BOT: 5’-AGCTTCAAAAACGGACAGGATGAATTTGACAACTCGAGTTGTCAAATTCATCCTGTCCGG-3’

ShR-RHOU-2

TOP: 5’-GATCCCAGTCGGATCTCAGAGAAGATCTCGAGATCTTCTCTGAGATCCGACTGTTTTTGA-3’

BOT: 5’-AGCTTCAAAAACAGTCGGATCTCAGAGAAGATCTCGAGATCTTCTCTGAGATCCGACTGG-3’

ShR-RHOU-3

TOP: 5’-GATCCCATCGTCGCTGGCATTCAATACTCGAGCATCGTCGCTGGCATTCAATATTTTTGA-3’

BOT: 5’-AGCTTCAAAAACATCGTCGCTGGCATTCAATACTCGAGCATCGTCGCTGGCATTCAATAG-3’


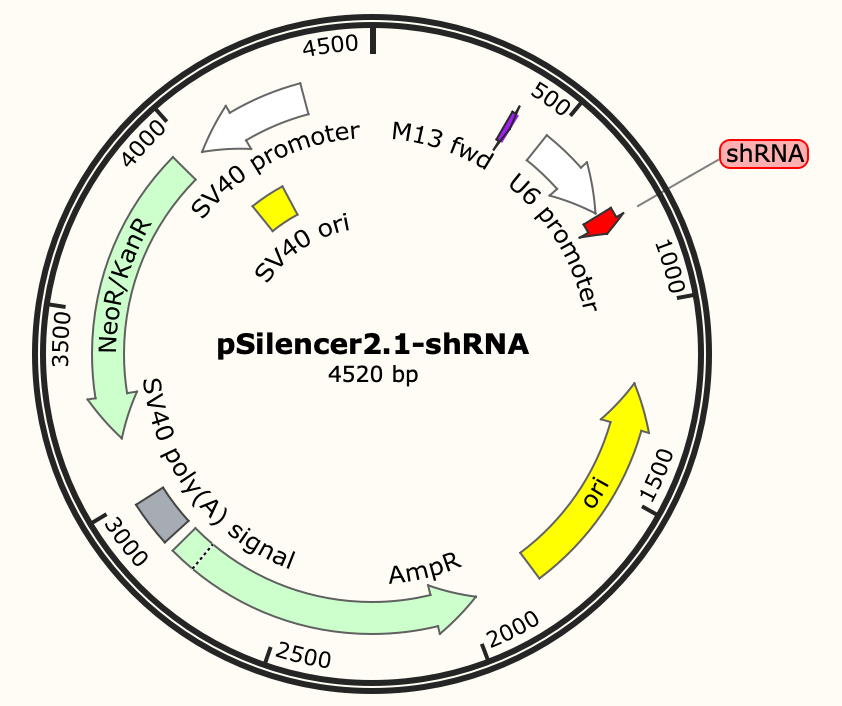


**Figure 2. The shRNA plasmid map.**
