# Supplementary material for: The novel antitumor compound clinopodiside A induces cytotoxicity via autophagy mediated by the signaling of BLK and RasGRP2 in T24 bladder cancer cells
Source: Front Pharmacol. 2022 Sep 19;13:982860. doi: 10.3389/fphar.2022.982860 (PMC9527273; doi:10.3389/fphar.2022.982860)
Supplement: Supplementary file 3 [file DataSheet1.ZIP › Original results/Figure 3A/clinopodiside A.pdf]

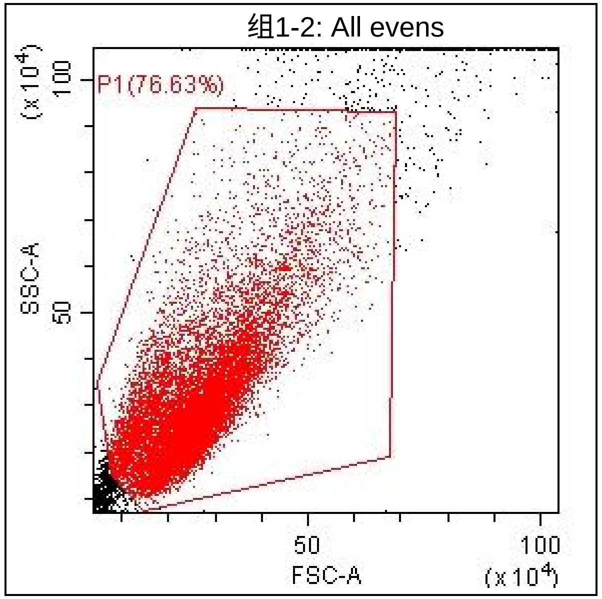


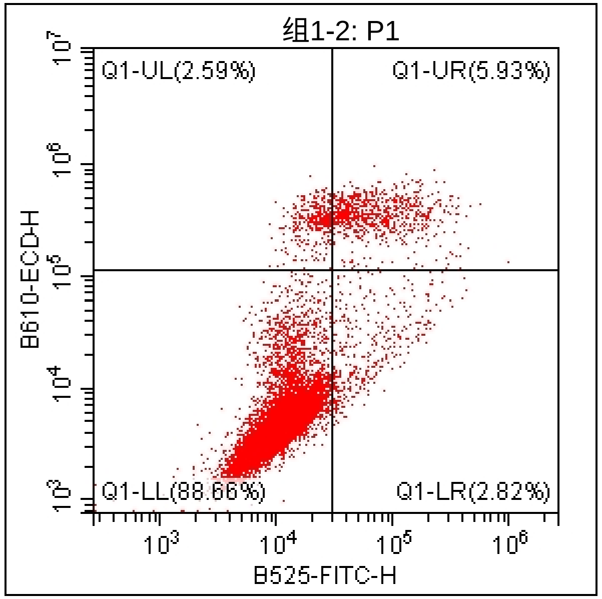


Tube Name: 组1-2

Sample ID: Clinopodiside A

| Population | Events | % Total | % Parent |
|---|---|---|---|
| All Events | 17481 | 100.00% | 100.00% |
| P1 | 13396 | 76.63% | 76.63% |
| Q1-UR | 794 | 4.54% | 5.93% |
| Q1-UL | 347 | 1.99% | 2.59% |
| Q1-LL | 11877 | 67.94% | 88.66% |
| Q1-LR | 378 | 2.16% | 2.82% |
