# Supplementary figures and images for: The novel antitumor compound clinopodiside A induces cytotoxicity via autophagy mediated by the signaling of BLK and RasGRP2 in T24 bladder cancer cells
Source: Front Pharmacol. 2022 Sep 19;13:982860. doi: 10.3389/fphar.2022.982860 (PMC9527273; doi:10.3389/fphar.2022.982860)

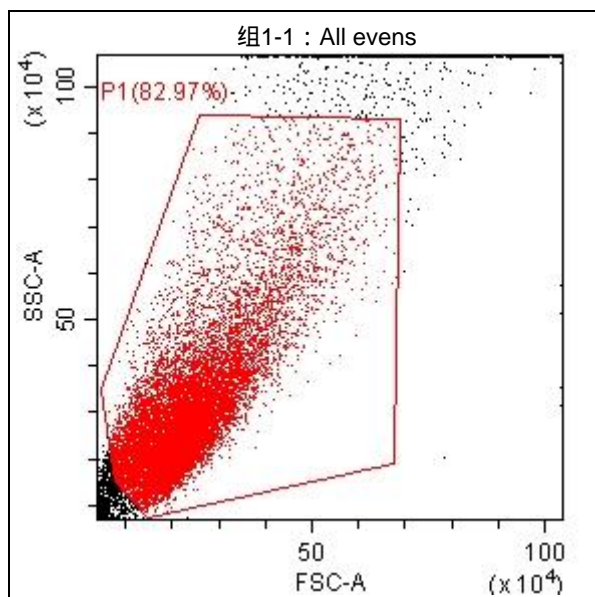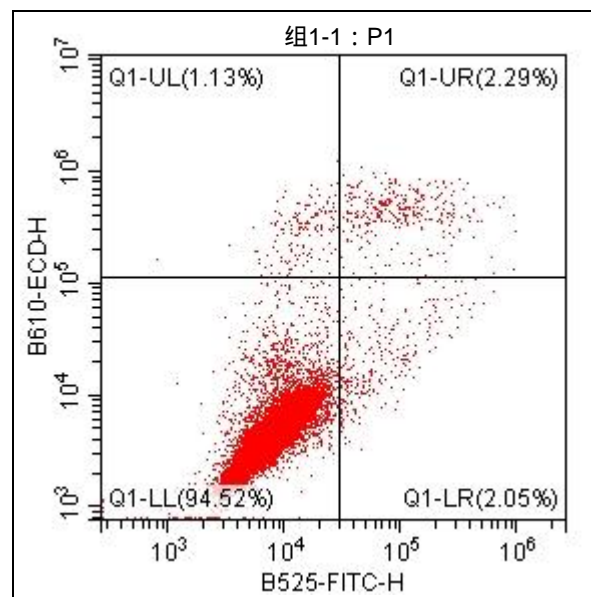

Tube Name: 组1-1

Sample ID: Control

| Population   | Events | % Total | % Parent |
|--------------|--------|---------|----------|
| ● All Events | 16611  | 100.00% | 100.00%  |
| ● P1         | 13782  | 82.97%  | 82.97%   |
| ⊗ Q1-UR      | 316    | 1.90%   | 2.29%    |
| ⊗ Q1-UL      | 156    | 0.94%   | 1.13%    |
| ⊗ Q1-LL      | 13027  | 78.42%  | 94.52%   |
| ⊗ Q1-LR      | 283    | 1.70%   | 2.05%    |

Supplement: Supplementary file 3 [file DataSheet1.ZIP › Original results/Figure 3A/Control.pdf]

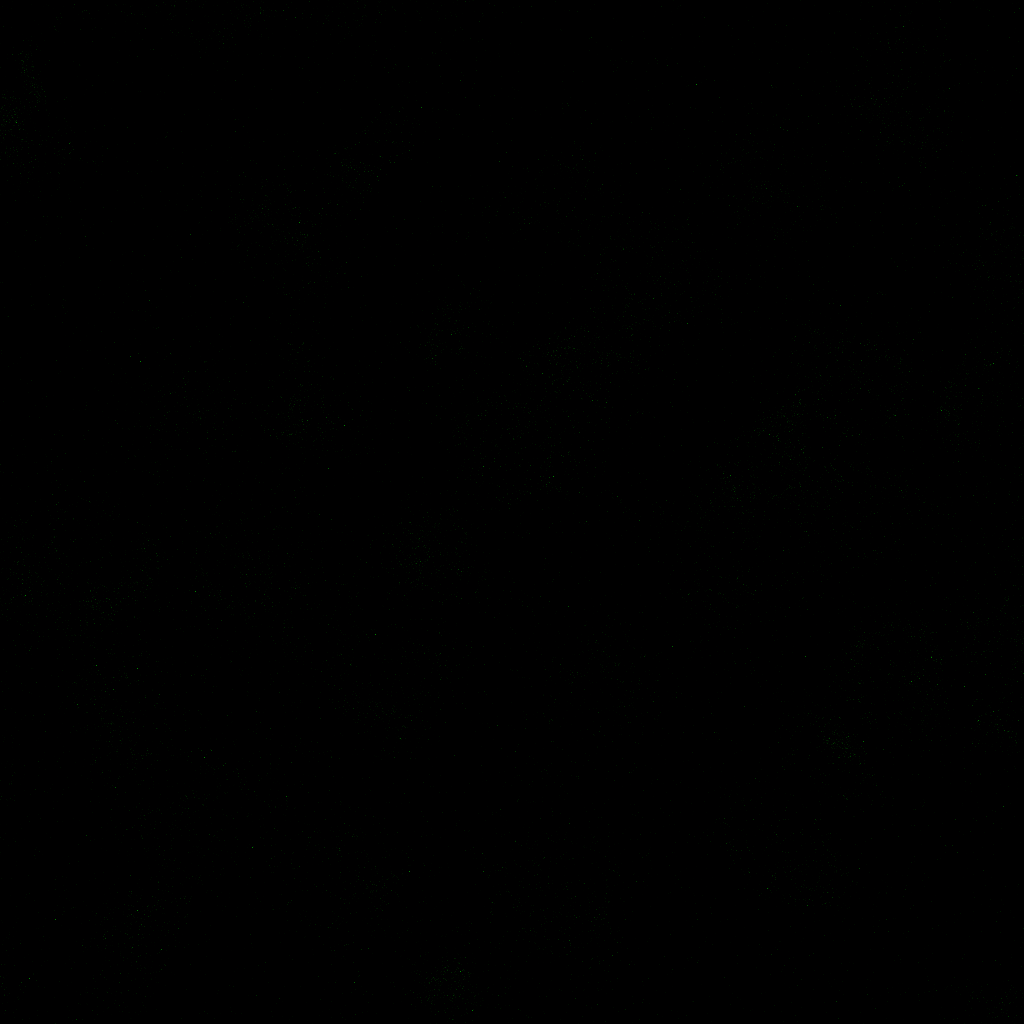

Supplement: Supplementary file 3 [file DataSheet1.ZIP › Original results/Figure 3D/Clinopodiside 0h/0h 600-1_C002T001.tif]

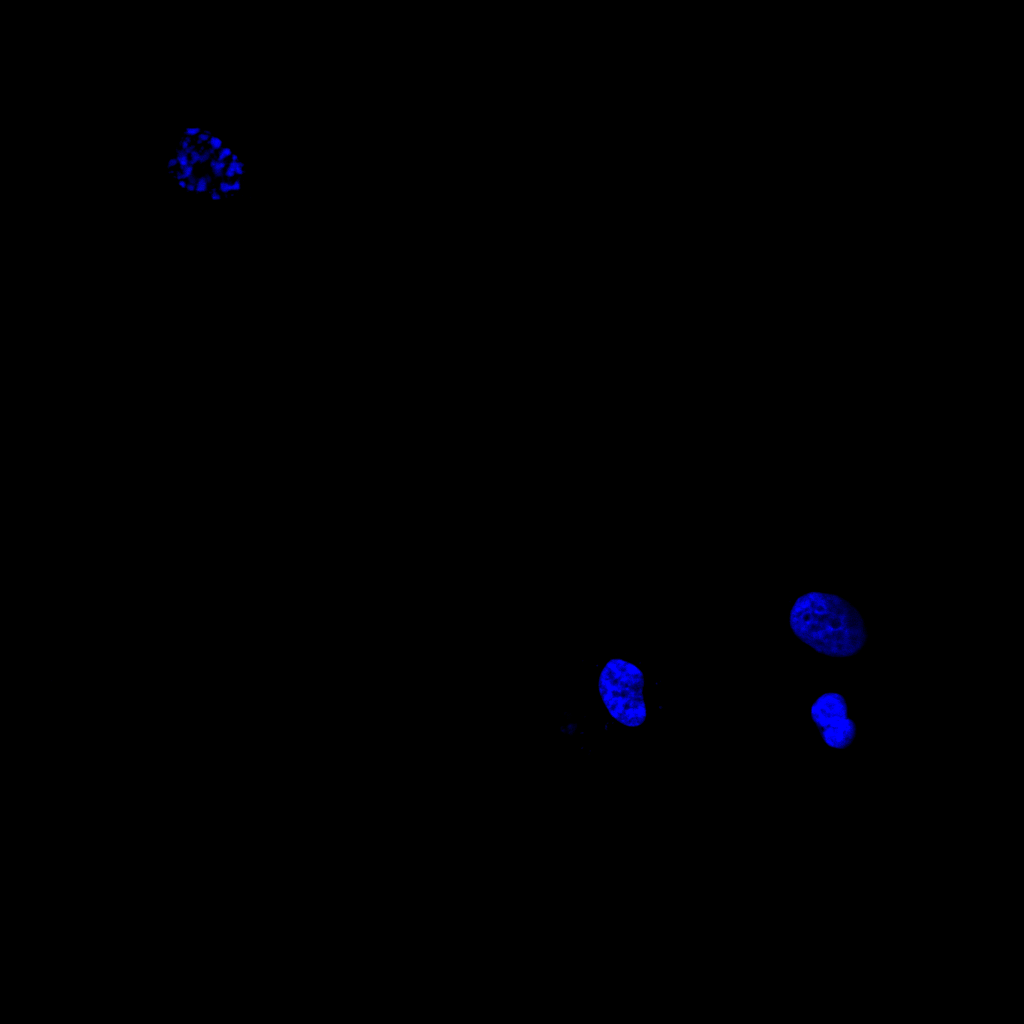

Supplement: Supplementary file 3 [file DataSheet1.ZIP › Original results/Figure 3D/Clinopodiside 0h/0h 600-2_C001T001.tif]

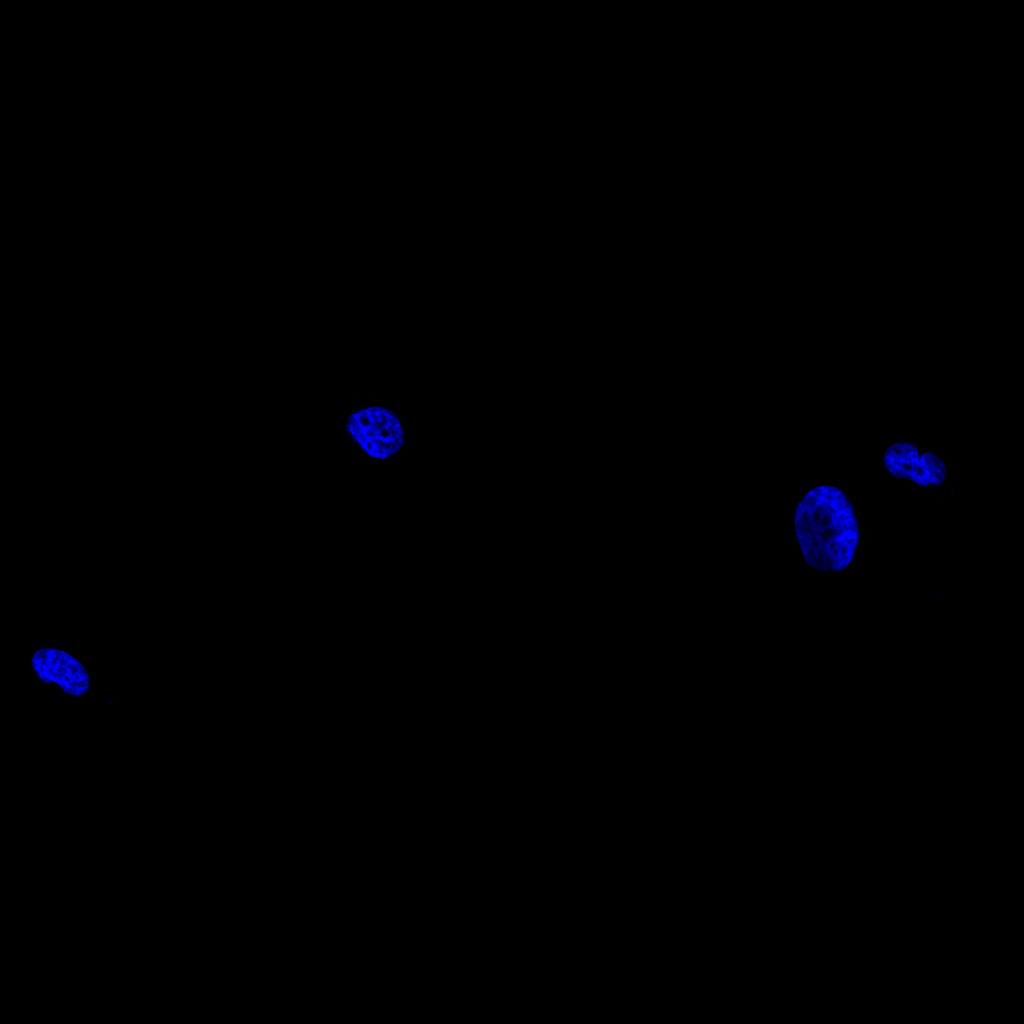

Supplement: Supplementary file 3 [file DataSheet1.ZIP › Original results/Figure 3D/Clinopodiside 4h/8h 600-2_C001T001.tif]

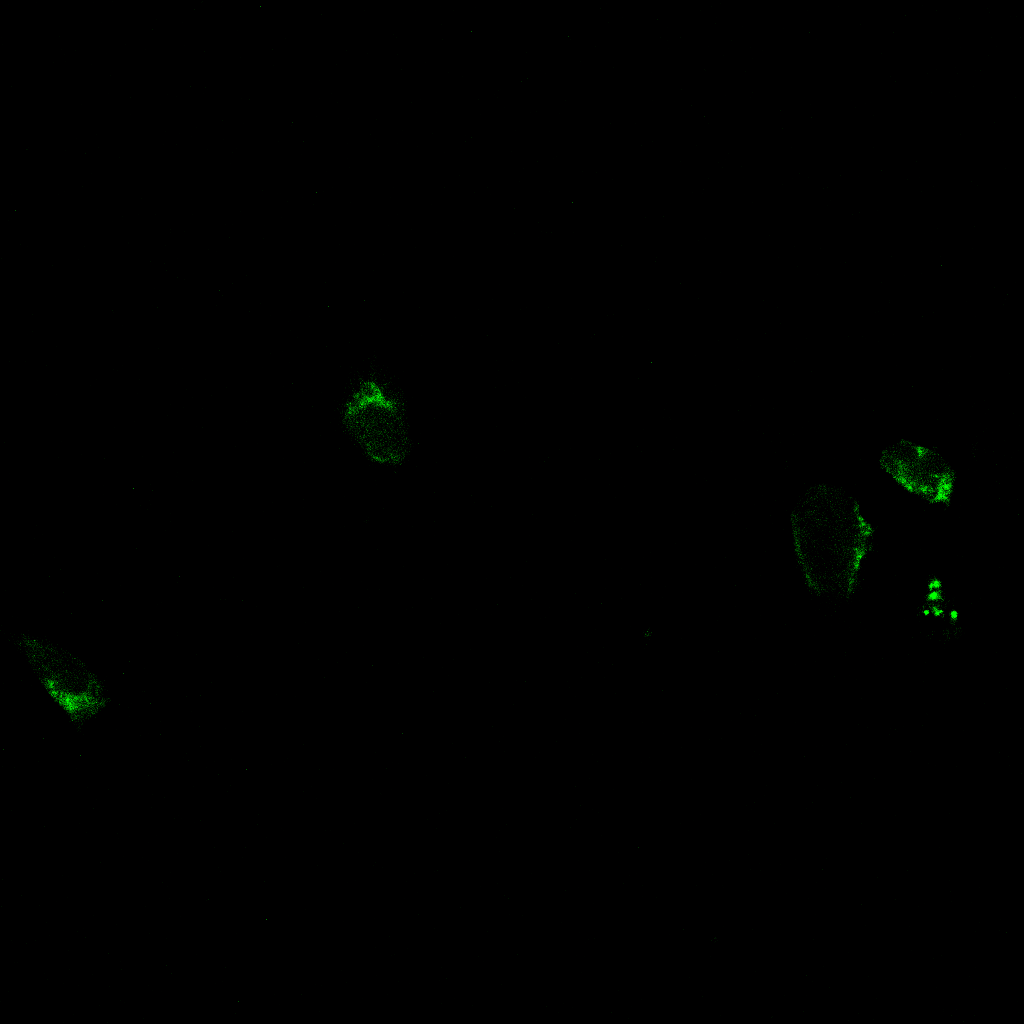

Supplement: Supplementary file 3 [file DataSheet1.ZIP › Original results/Figure 3D/Clinopodiside 4h/8h 600-2_C002T001.tif]

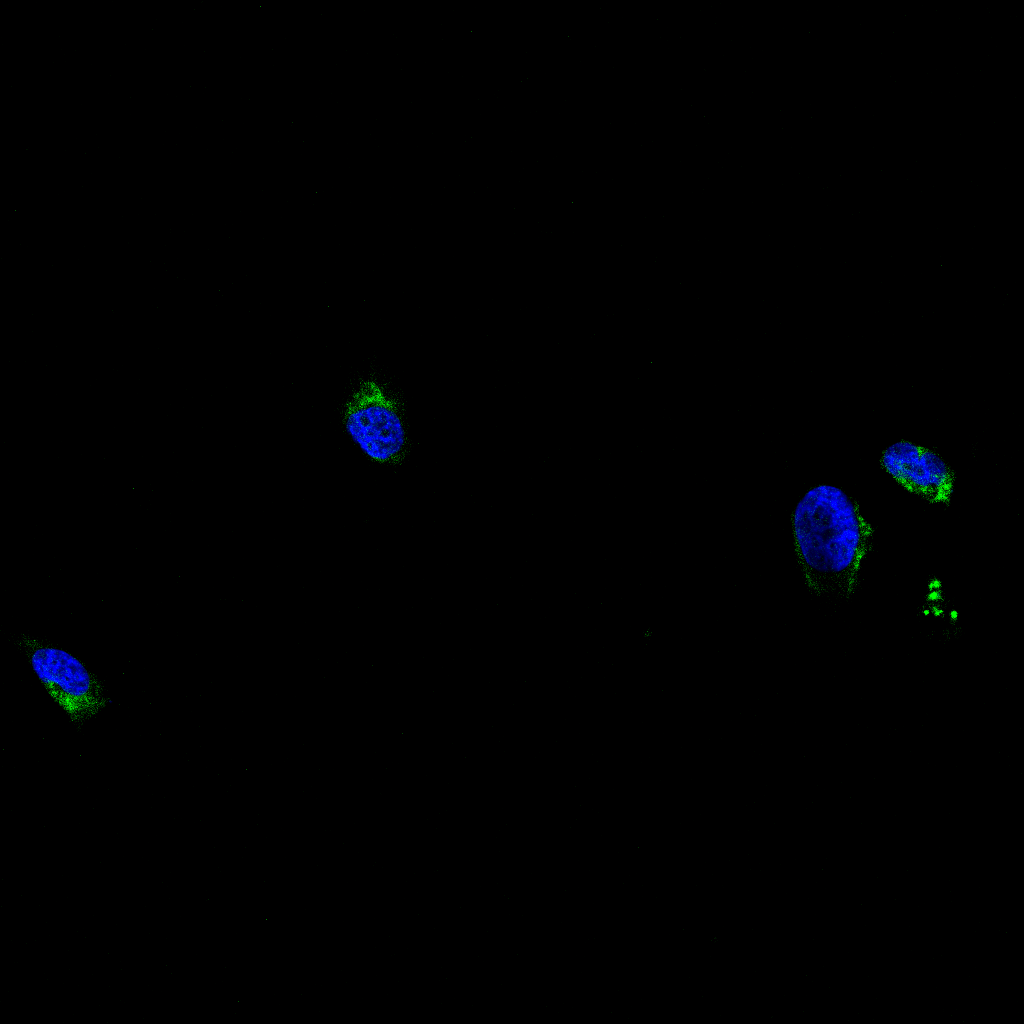

Supplement: Supplementary file 3 [file DataSheet1.ZIP › Original results/Figure 3D/Clinopodiside 4h/8h 600-2_T001.tif]

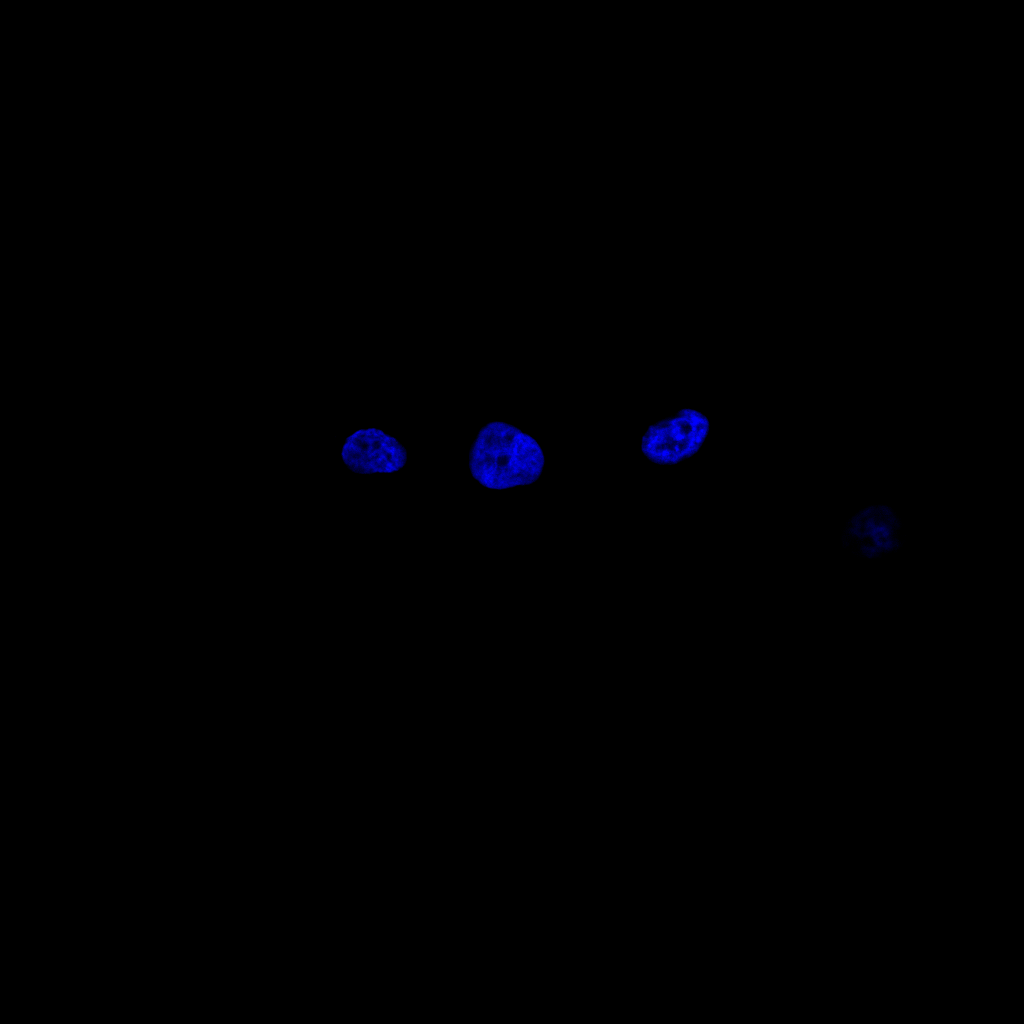

Supplement: Supplementary file 3 [file DataSheet1.ZIP › Original results/Figure 3D/Clinopodiside 8h/12h 600-1_C001T001.tif]

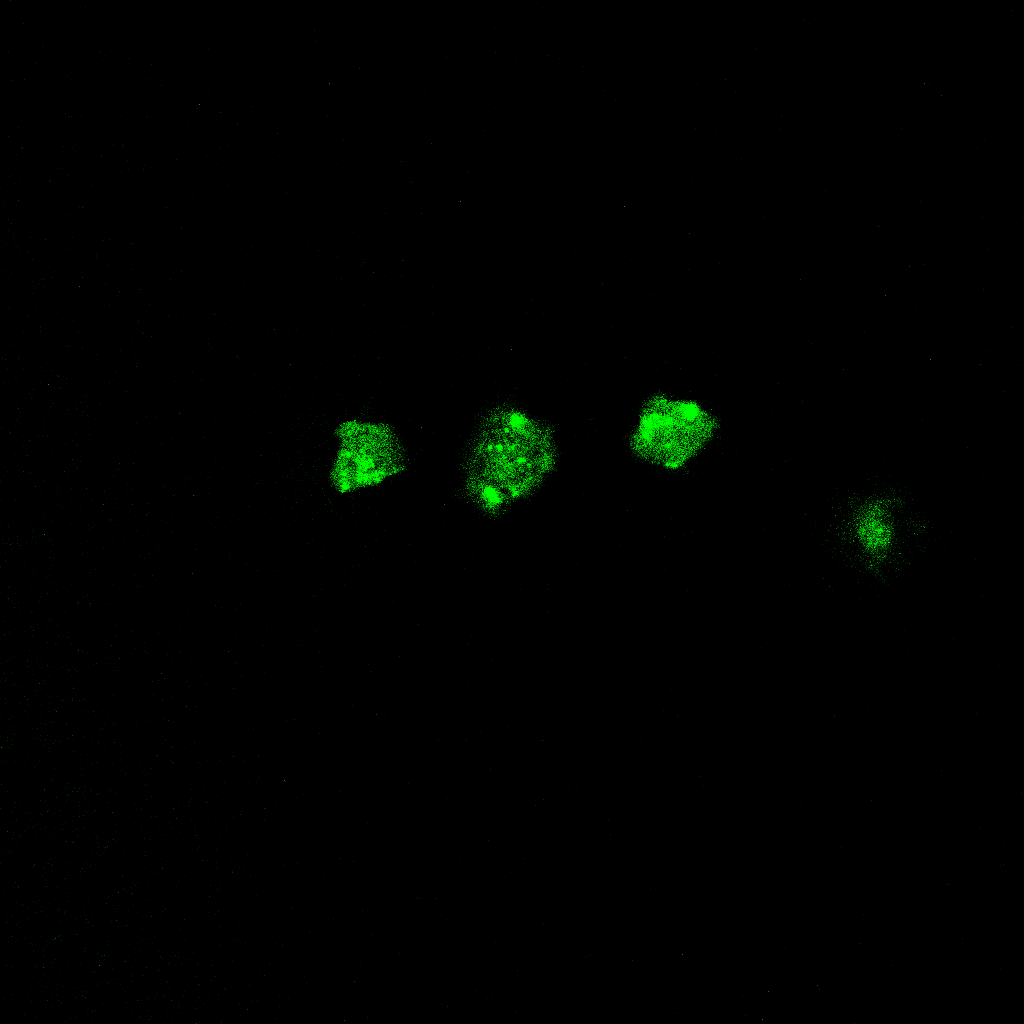

Supplement: Supplementary file 3 [file DataSheet1.ZIP › Original results/Figure 3D/Clinopodiside 8h/12h 600-1_C002T001.tif]

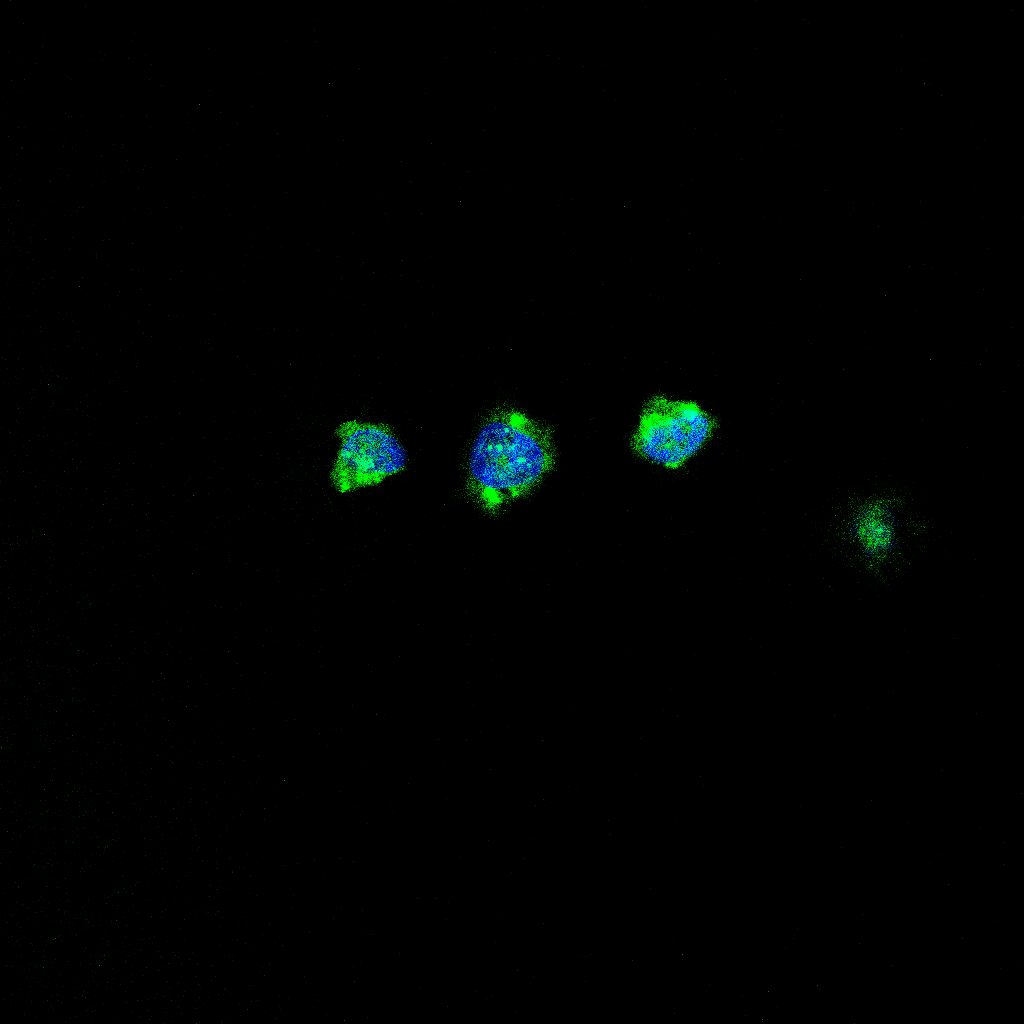

Supplement: Supplementary file 3 [file DataSheet1.ZIP › Original results/Figure 3D/Clinopodiside 8h/12h 600-1_T001.tif]

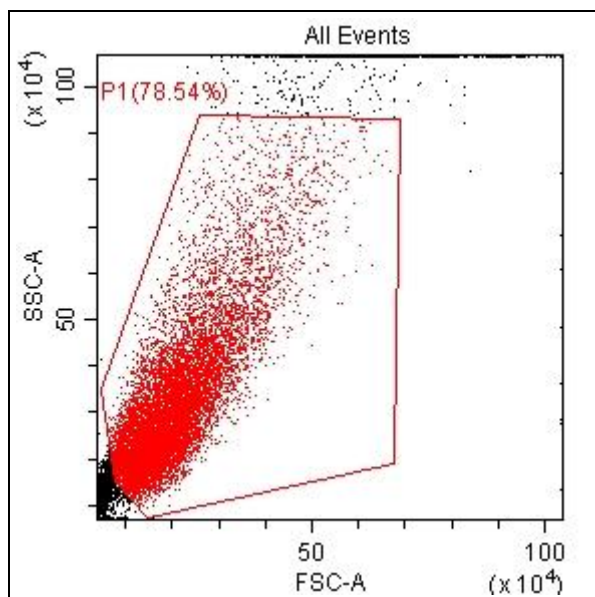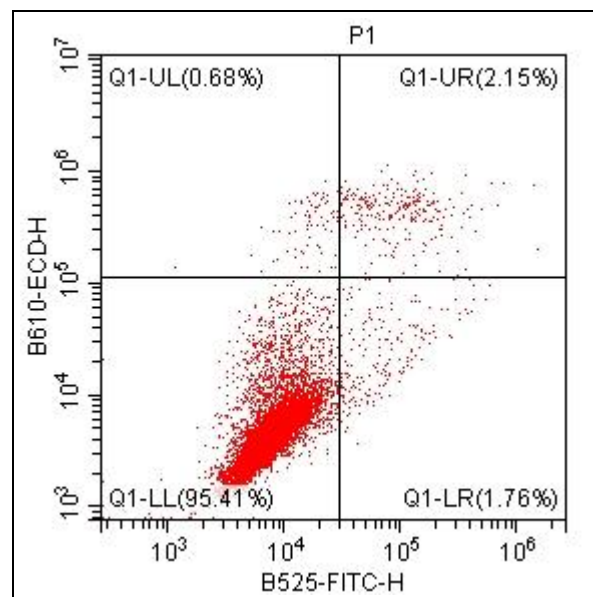

Tube Name:

Sample ID:

| Population   | Events | % Total | % Parent |
|--------------|--------|---------|----------|
| ● All Events | 14095  | 100.00% | 100.00%  |
| ● P1         | 11070  | 78.54%  | 78.54%   |
| ⊗ Q1-UR      | 238    | 1.69%   | 2.15%    |
| ⊗ Q1-UL      | 75     | 0.53%   | 0.68%    |
| ⊗ Q1-LL      | 10562  | 74.93%  | 95.41%   |
| ⊗ Q1-LR      | 195    | 1.38%   | 1.76%    |

Supplement: Supplementary file 3 [file DataSheet1.ZIP › Original results/Figure 7C/A.pdf]

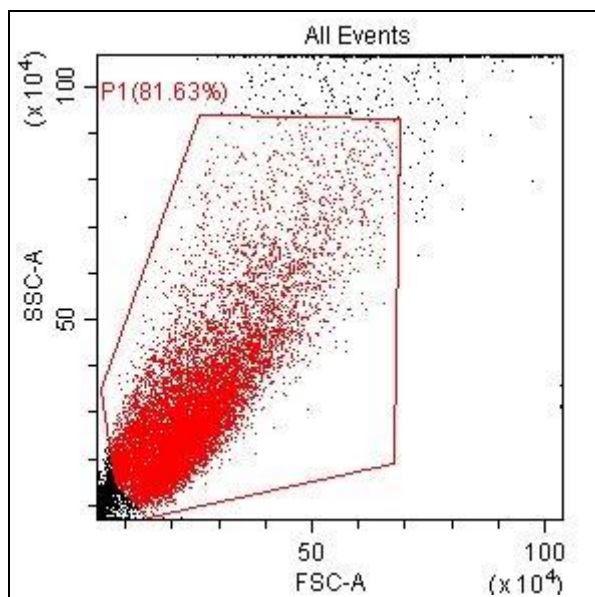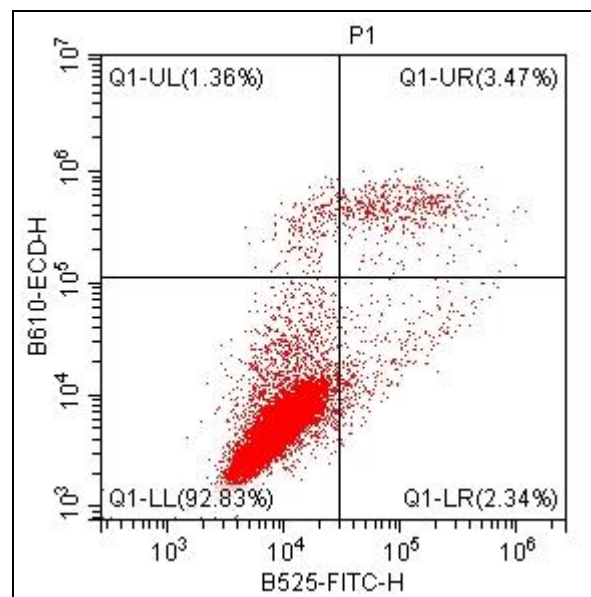

Tube Name:

Sample ID:

| Population   | Events | % Total | % Parent |
|--------------|--------|---------|----------|
| ● All Events | 16397  | 100.00% | 100.00%  |
| ● P1         | 13385  | 81.63%  | 81.63%   |
| ⊗ Q1-UR      | 465    | 2.84%   | 3.47%    |
| ⊗ Q1-UL      | 182    | 1.11%   | 1.36%    |
| ⊗ Q1-LL      | 12425  | 75.78%  | 92.83%   |
| ⊗ Q1-LR      | 313    | 1.91%   | 2.34%    |

Supplement: Supplementary file 3 [file DataSheet1.ZIP › Original results/Figure 7C/B.pdf]

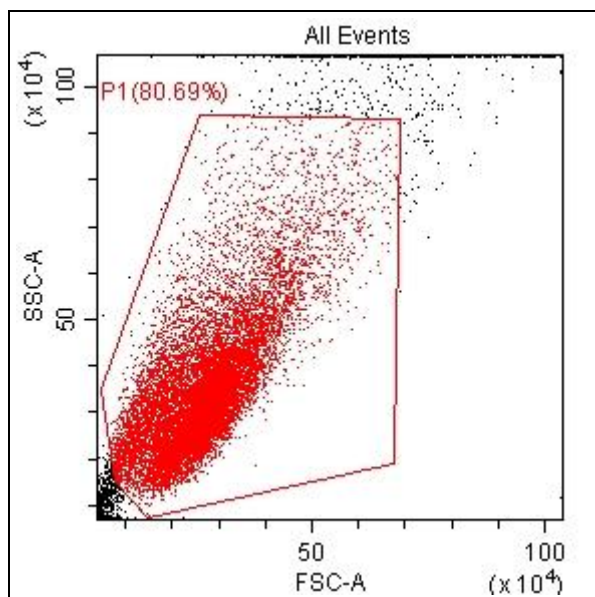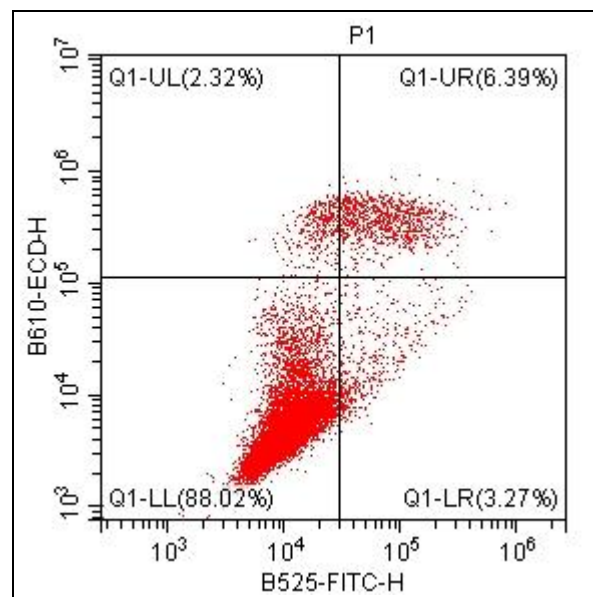

Tube Name:

Sample ID:

| Population   | Events | % Total | % Parent |
|--------------|--------|---------|----------|
| ● All Events | 16561  | 100.00% | 100.00%  |
| ● P1         | 13363  | 80.69%  | 80.69%   |
| ⊗ Q1-UR      | 854    | 5.16%   | 6.39%    |
| ⊗ Q1-UL      | 310    | 1.87%   | 2.32%    |
| ⊗ Q1-LL      | 11762  | 71.02%  | 88.02%   |
| ⊗ Q1-LR      | 437    | 2.64%   | 3.27%    |

Supplement: Supplementary file 3 [file DataSheet1.ZIP › Original results/Figure 7C/C.pdf]

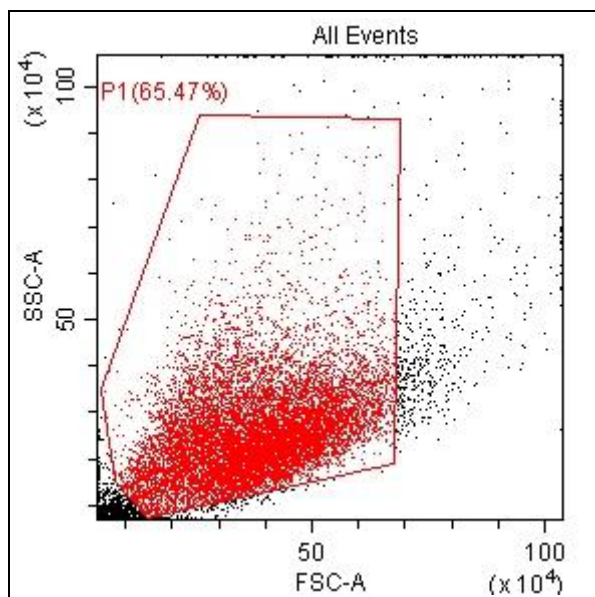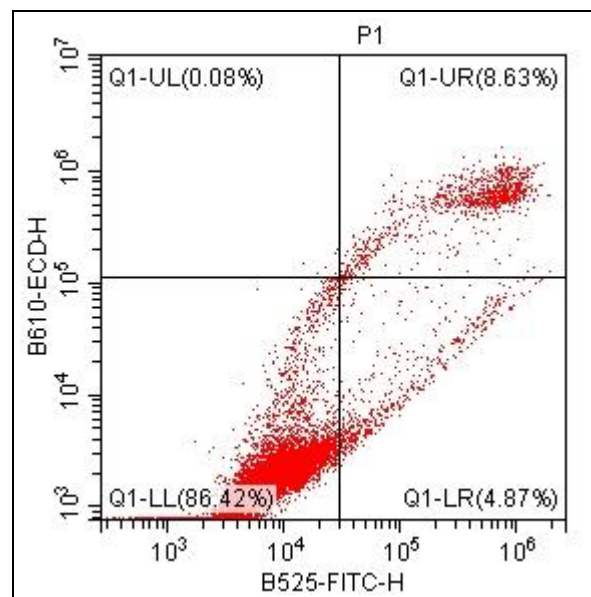

Tube Name:

Sample ID:

| Population   | Events | % Total | % Parent |
|--------------|--------|---------|----------|
| ● All Events | 17299  | 100.00% | 100.00%  |
| ● P1         | 11325  | 65.47%  | 65.47%   |
| ⊗ Q1-UR      | 977    | 5.65%   | 8.63%    |
| ⊗ Q1-UL      | 9      | 0.05%   | 0.08%    |
| ⊗ Q1-LL      | 9787   | 56.58%  | 86.42%   |
| ⊗ Q1-LR      | 552    | 3.19%   | 4.87%    |

Supplement: Supplementary file 3 [file DataSheet1.ZIP › Original results/Figure 7C/D.pdf]

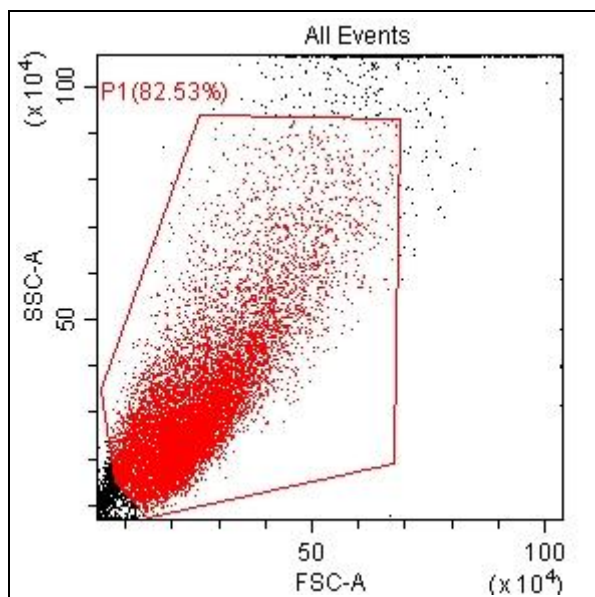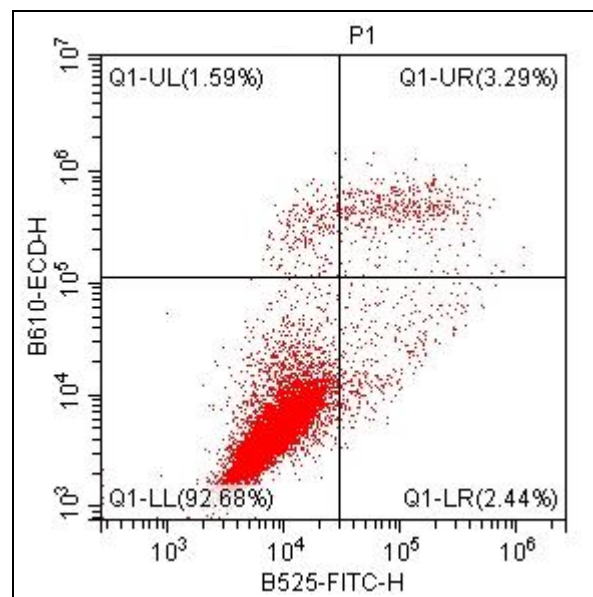

Tube Name:

Sample ID:

| Population   | Events | % Total | % Parent |
|--------------|--------|---------|----------|
| ● All Events | 15836  | 100.00% | 100.00%  |
| ● P1         | 13070  | 82.53%  | 82.53%   |
| ⊗ Q1-UR      | 430    | 2.72%   | 3.29%    |
| ⊗ Q1-UL      | 208    | 1.31%   | 1.59%    |
| ⊗ Q1-LL      | 12113  | 76.49%  | 92.68%   |
| ⊗ Q1-LR      | 319    | 2.01%   | 2.44%    |

Supplement: Supplementary file 3 [file DataSheet1.ZIP › Original results/Figure 7C/E.pdf]

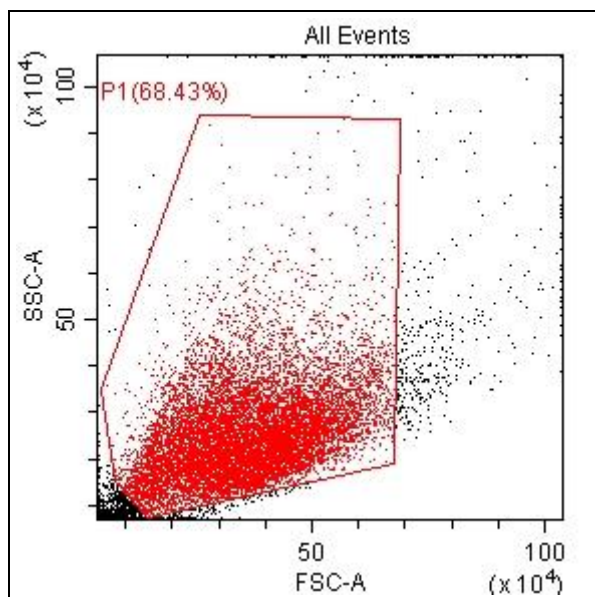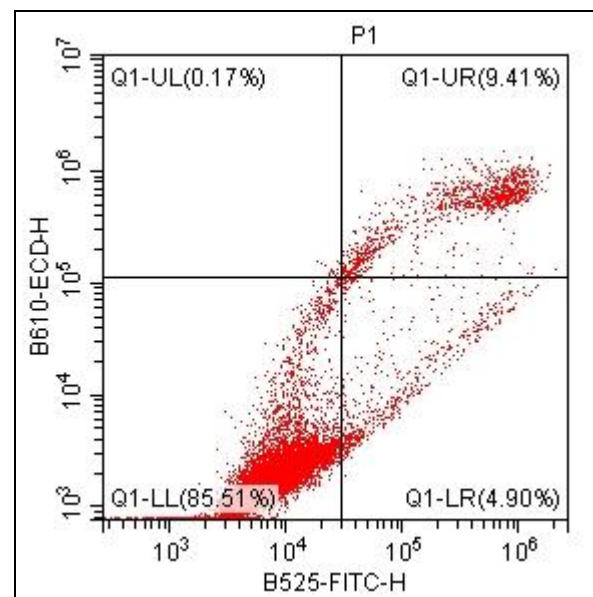

Tube Name:

Sample ID:

| Population   | Events | % Total | % Parent |
|--------------|--------|---------|----------|
| ● All Events | 16956  | 100.00% | 100.00%  |
| ● P1         | 11603  | 68.43%  | 68.43%   |
| ⊗ Q1-UR      | 1092   | 6.44%   | 9.41%    |
| ⊗ Q1-UL      | 20     | 0.12%   | 0.17%    |
| ⊗ Q1-LL      | 9922   | 58.52%  | 85.51%   |
| ⊗ Q1-LR      | 569    | 3.36%   | 4.90%    |

Supplement: Supplementary file 3 [file DataSheet1.ZIP › Original results/Figure 7C/F.pdf]

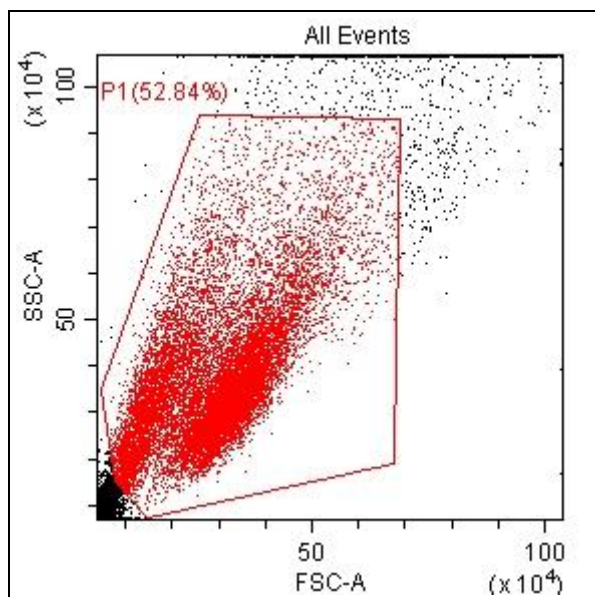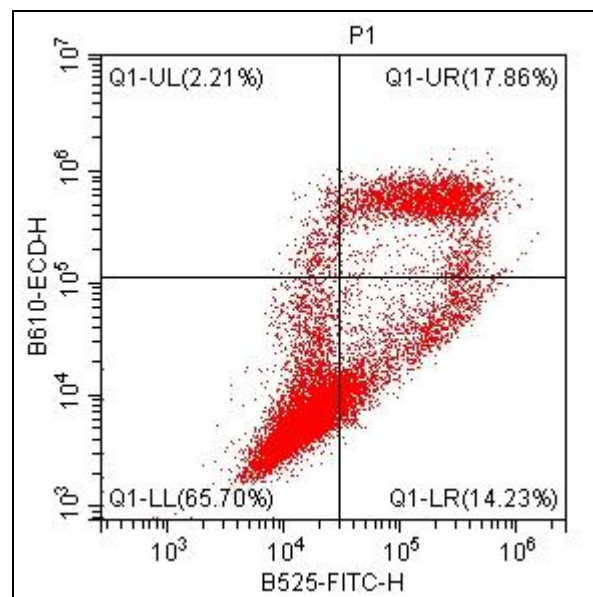

Tube Name:

Sample ID:

| Population   | Events | % Total | % Parent |
|--------------|--------|---------|----------|
| ● All Events | 27485  | 100.00% | 100.00%  |
| ● P1         | 14522  | 52.84%  | 52.84%   |
| ⊗ Q1-UR      | 2593   | 9.43%   | 17.86%   |
| ⊗ Q1-UL      | 321    | 1.17%   | 2.21%    |
| ⊗ Q1-LL      | 9541   | 34.71%  | 65.70%   |
| ⊗ Q1-LR      | 2067   | 7.52%   | 14.23%   |

Supplement: Supplementary file 3 [file DataSheet1.ZIP › Original results/Figure 7C/G.pdf]

Figure 3B

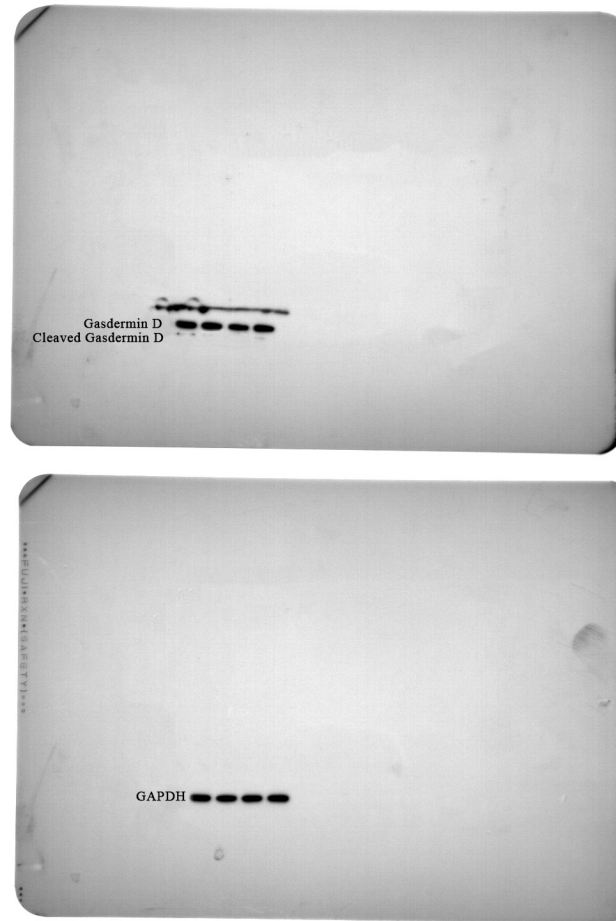

Figure 3C

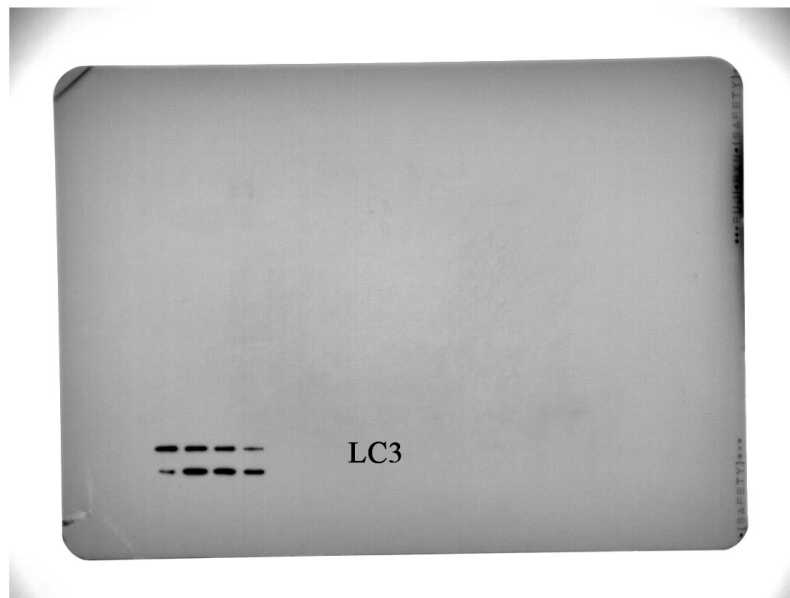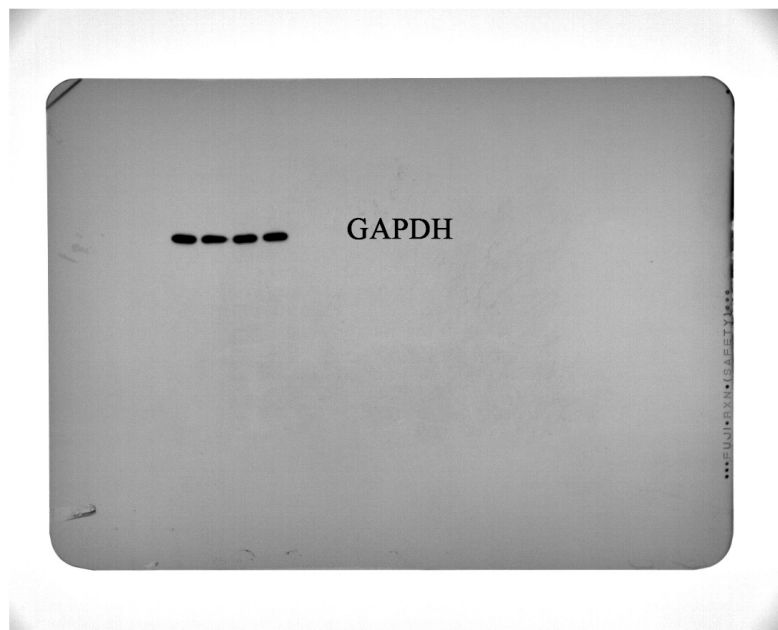

Figure 3E

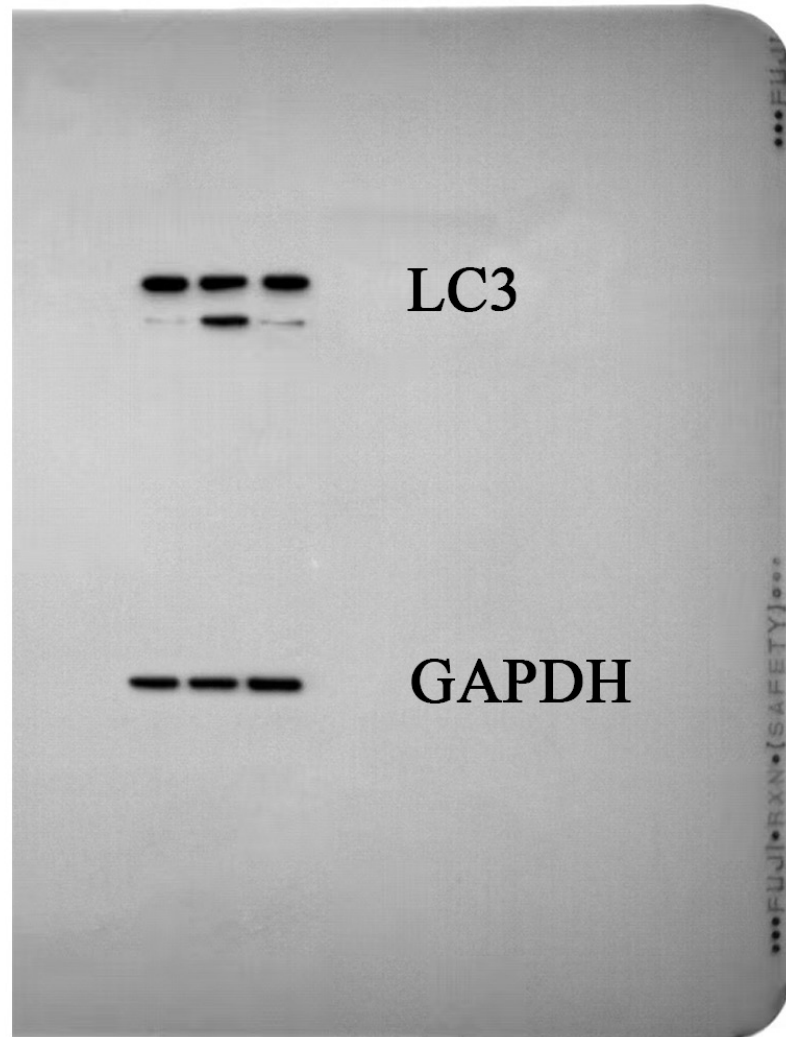

Figure 5A

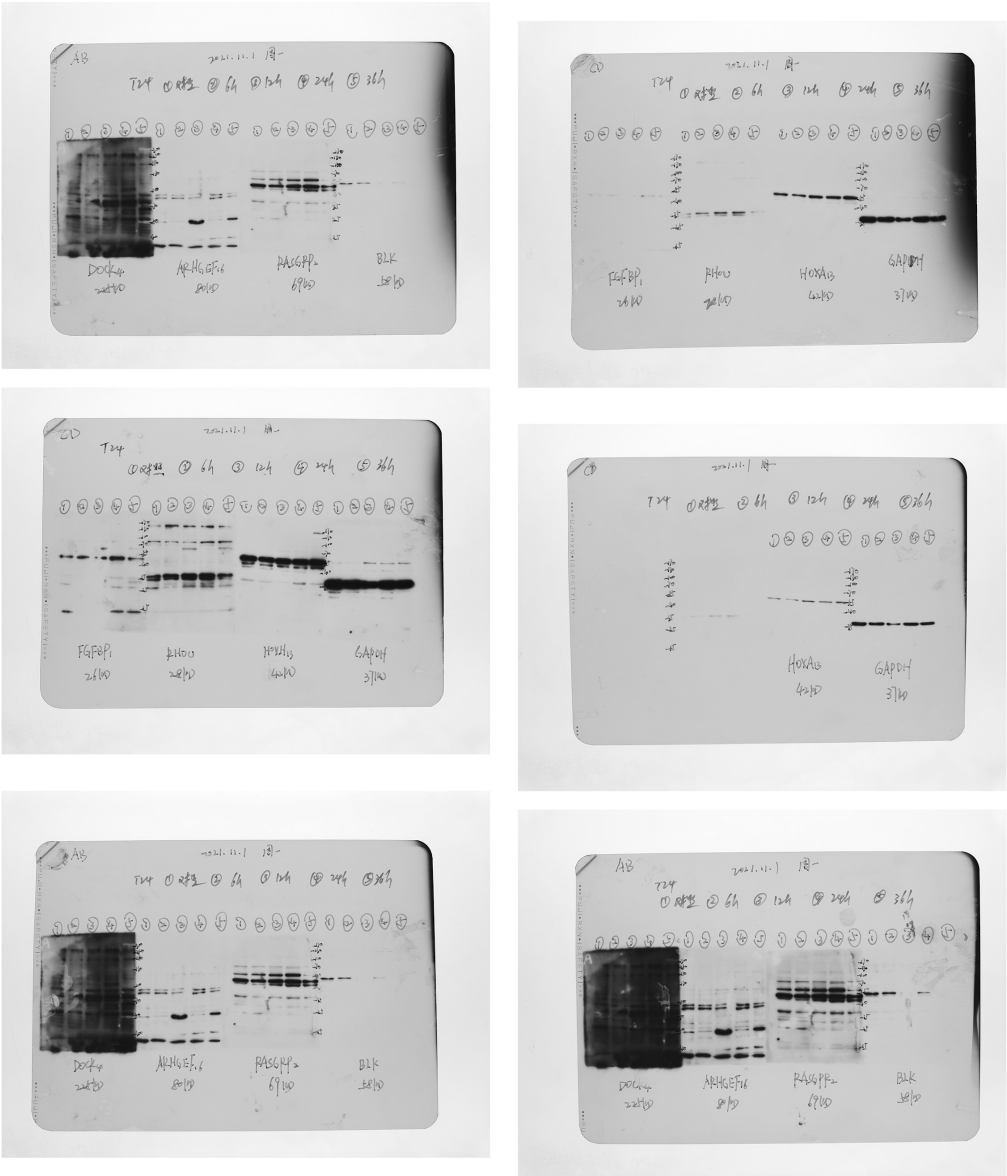

Figure 5B

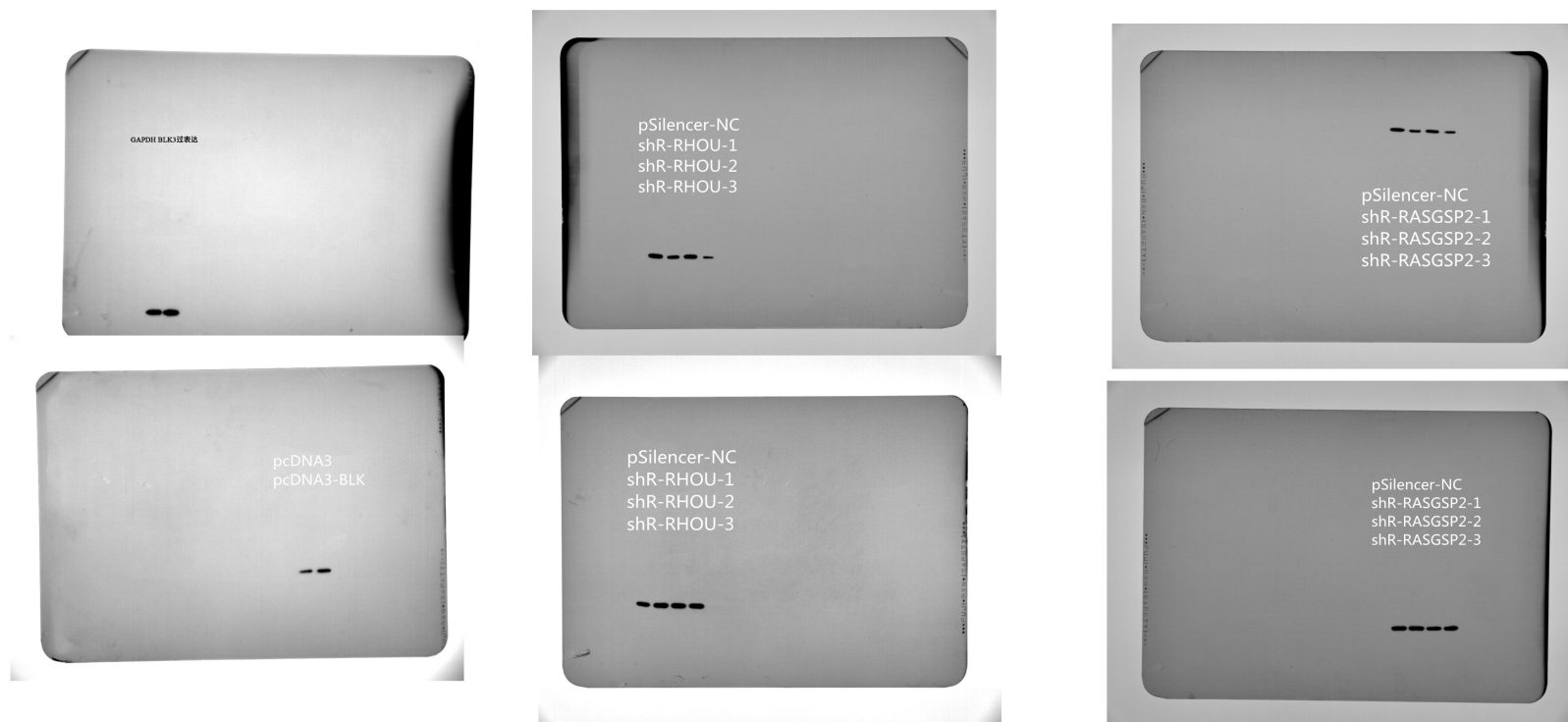

Figure 5C

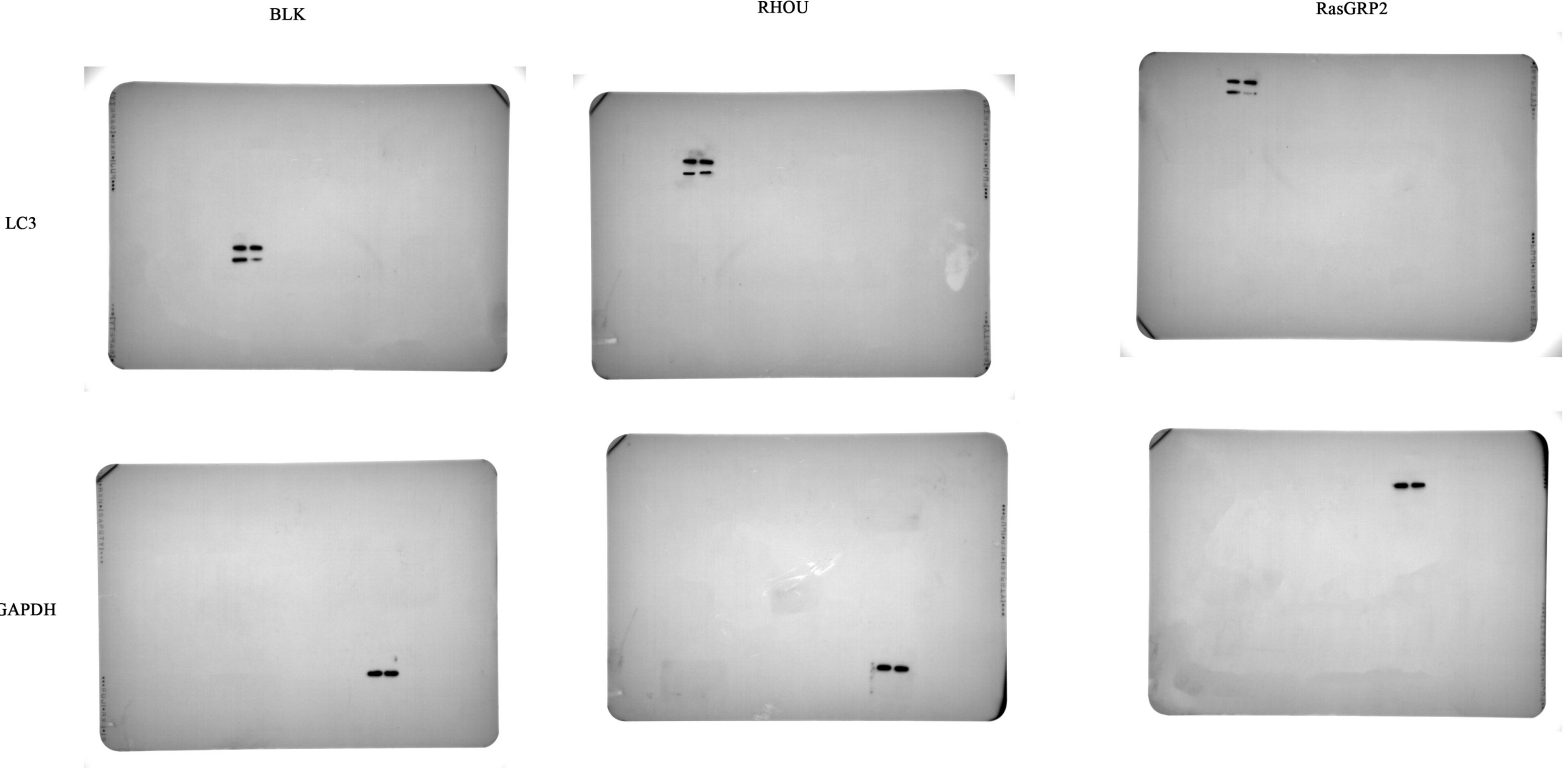

Figure 5E

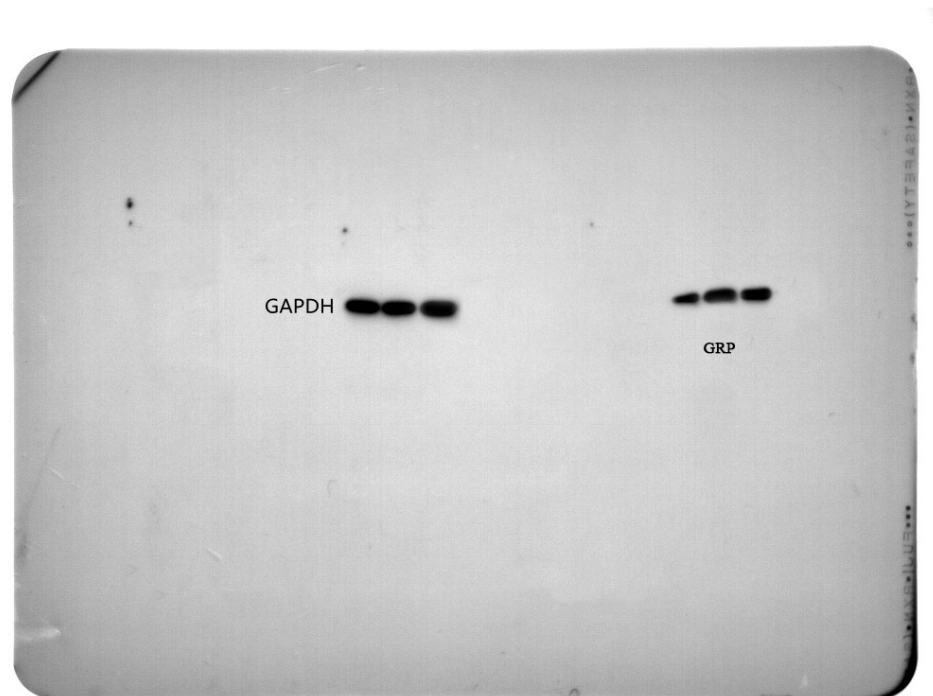

Figure 5F

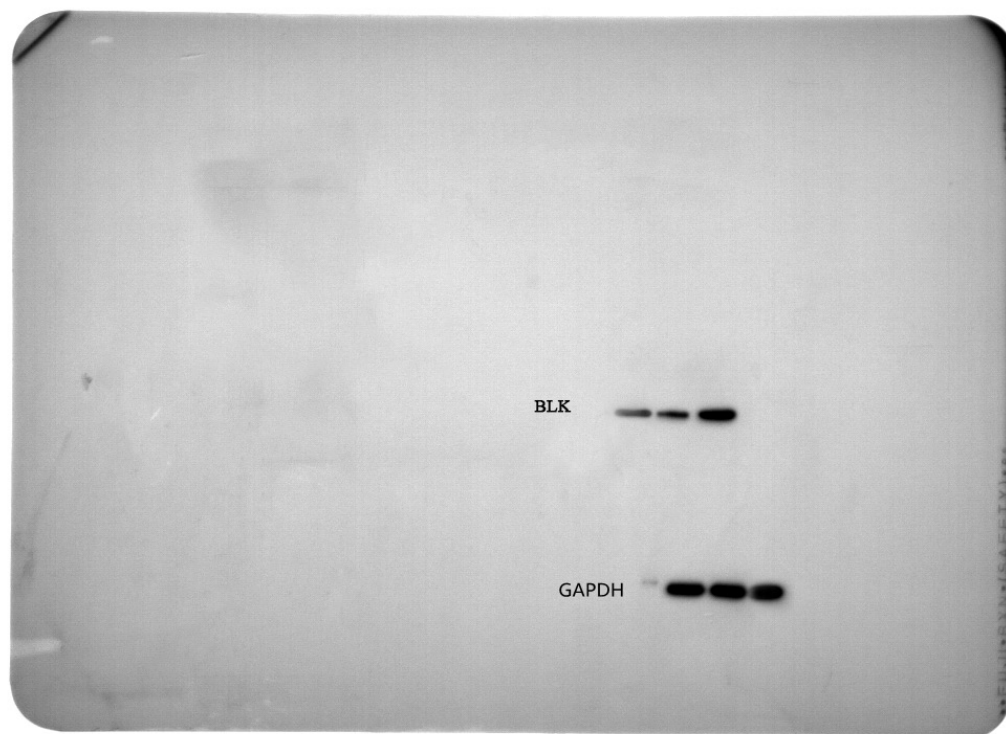

Figure 7A

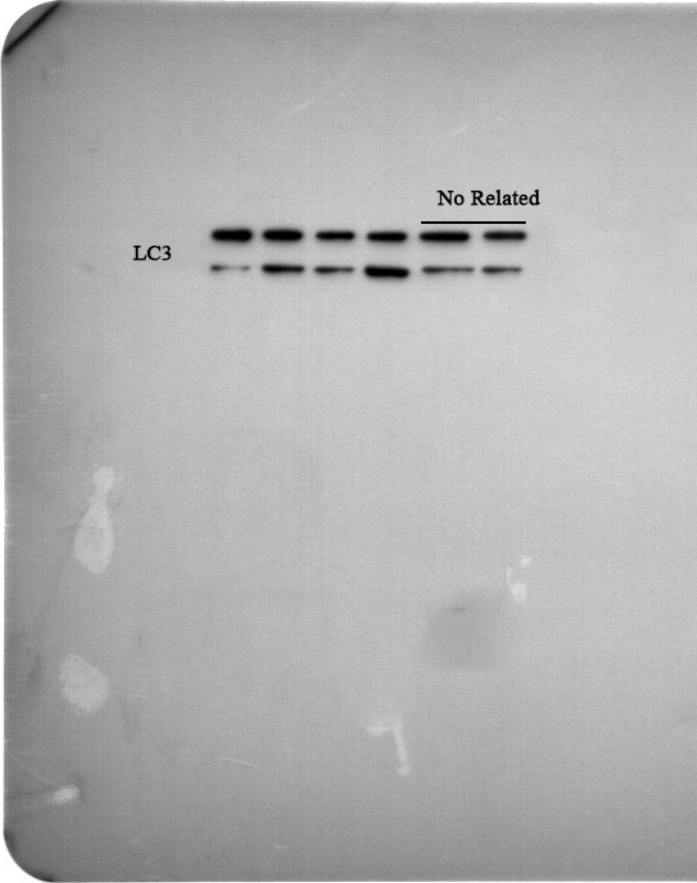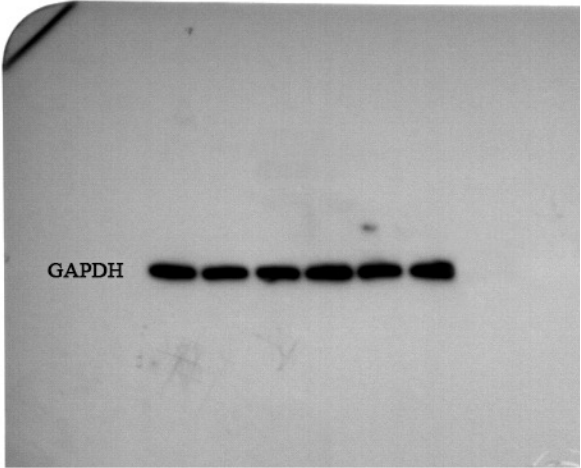

Supplement: Supplementary file 3 [file DataSheet1.ZIP › Original results/Western blot original figures.pdf]
